# *Trichoderma reesei* Nsd3 transcription factor: pleiotropic roles in development, stress response, secondary metabolism, and cellulase production

**DOI:** 10.1128/aem.00865-26

**Published:** 2026-09-03

**Authors:** David Batista Maués, Lucas Matheus Soares Pereira, Andrei Stecca Steindorff, Amanda Cristina Campos Antoniêto, Rafael Silva-Rocha, Marcelo Damário Gomes, Zachary A. Lewis, Roberto N. Silva

**Affiliations:** 1 Department of Biochemistry and Immunology, Ribeirão Preto Medical School, University of São Paulo 28133 , Ribeirão Preto, São Paulo, Brazil; 2 US Department of Energy Joint Genome Institute, Lawrence Berkeley National Laboratory 1666, Berkeley, California, USA; 3 ByMyCell Inova Simples LTDA, Ribeirão Preto, São Paulo, Brazil; 4 Department of Microbiology, University of Georgia 1355, Athens, Georgia, USA; 5 National Institute of Science and Technology in Human Pathogenic Fungi, São Paulo, Brazil; Chalmers tekniska hogskola AB, Gothenburg, Sweden

**Keywords:** *Trichoderma reesei*, cellulase, stress response, Nsd3, systems biology, ATAC-Seq

## Abstract

*Trichoderma reesei* is known for its ability to secrete high amounts of cellulases, enzymes of fundamental importance in generating products from lignocellulosic biomass. Diverse signaling pathways and transcription factors (TFs) control the cellulolytic repertoire in *T. reesei* to ensure correct adaptation to the environment. Here, we analyzed RNA-Seq data and identified a new potential regulator of cellulase production in *T. reesei*: a novel TF named Nsd3, a homolog of NsdC from *Aspergilli*. Deletion of *nsd3* reduced vegetative growth and conidiation on solid medium. Phenotypic characterization of the Δ*nsd3* strain showed that it is more sensitive to osmotic stress, but more resistant to cell wall and oxidative stresses. Our results showed that Nsd3 is a repressor of cellulase expression by directly regulating key genes in the cellulolytic pathway, an unreported role for this TF in fungi. Loss of *nsd3* leads to a faster and more robust induction of cellulolytic genes, and higher cellulase and hemicellulase activities. Transcriptional profiling by RNA-Seq, chromatin accessibility profiling by ATAC-Seq, and protein-DNA interaction assays showed that sugar transporters are important targets of Nsd3 during cellulase expression regulation. Combined with microscopy and gene expression analyses, the ATAC-Seq data also highlighted Nsd3 as a central regulator of cell wall remodeling and organization. Furthermore, the transcriptomics also showed that Nsd3 regulates genes involved in secondary metabolism. These results showed that Nsd3 regulates several physiological processes and provide novel insights into the regulatory system of cellulases in *T. reesei* that can be used in the design of high-performance strains for biorefinery.

**IMPORTANCE:**

*Trichoderma reesei* is a key player in the production of hydrolytic enzymes for the degradation of lignocellulose biomass, and transcription factors are important targets for genetic engineering to construct cellulase-hyperproducing strains. Here, we identified the transcription factor Nsd3 and characterized its role as a regulator of cellulase production in *T. reesei*. We applied two powerful genomics methods (transcriptome sequencing and chromatin accessibility sequencing) to unravel the global role of Nsd3 and its regulatory mechanism. Nsd3 participates in various biological processes in *T. reesei*, including cell wall remodeling, calcium metabolism, and secondary metabolism, in addition to regulating the expression of sugar transporters. Protein-DNA interaction assays demonstrate that Nsd3 acts through important genes to regulate cellulase expression, including ace4, crt1, stp1, and cel1b. Our study provides mechanistic insights about how Nsd3 regulates diverse physiological processes in *T. reesei*. This work also applied ATAC-Seq for the first time to study chromatin accessibility in *T. reesei*.

## INTRODUCTION

Filamentous fungi are important organisms for the degradation of lignocellulose biomass (LCB), the most abundant carbon source on Earth. In recent decades, the LCB has become a target in the biorefinery industry for the production of fine chemicals and generation of value-added products, such as biofuels (1). LCB degradation is carried out by a set of enzymes called cellulases, hemicellulases, and lignin-modifying enzymes that act synergistically to depolymerize the LCB and release fermentable sugars that can be converted to the desired bioproducts (2). The saprophytic fungus *Trichoderma reesei* is a key player in LCB degradation, due to its high ability to secrete high amounts of cellulases (up to 80 g/L), being used for decades for enzyme production on a commercial scale (3). The *T. reesei* holocellulolytic system comprises two cellobiohydrolases (Cel7a and Cel6a), which act at the ends of cellulose chains, releasing the disaccharide β-cellobiose; seven endoglucanases, which cleave the internal regions of cellulose chains; 11 β-glucosidases, which cleave cellobiose, releasing glucose; and several hemicellulases and other accessory proteins that help with LCB degradation. Cel7a is the most dominant enzyme, comprising about 70% of the *T. reesei* secretome (4, 5).

Cellulase production involves several complex mechanisms, from nutrient sensing to the appropriate secretion of these enzymes, integrating several signaling pathways that culminate in efficient production. In the presence of the complex polymers that make up the plant cell wall, fungi activate the expression of cellulases and hemicellulases, which are secreted to degrade this biomass into sugars that can be easily transported into the cell and then metabolized (6). The disaccharide sophorose, formed from cellobiose through a reaction called transglycosylation, is the most potent inducer of cellulase expression in *T. reesei* (7). So far, it is not clear how sophorose triggers the induction of cellulase expression. Some *T. reesei* β-glucosidases (extra- and intracellular) exhibit transglycosylation activity (8). Therefore, sophorose can be produced from cellobiose either inside or outside the cell. *T. reesei* sugar transporters Crt1 and Tr44175 are the only ones described so far that are capable of transporting sophorose, in addition to other cellooligosaccharides (9–11). Crt1 acts as a transceptor and is critical for cellulase expression. Deletion of *crt1* abolishes cellulase production in *T. reesei* (12, 13).

After the cell receives and transduces the cellulose signal, transcription factors (TFs) activate the expression of holocellulases, sugar transporters, and other proteins involved in biomass degradation. In *T. reesei*, the TF Xyr1 is the master regulator of biomass degradation, activating the expression of most of the hydrolytic genes (14). In contrast, the TF Cre1 is the main repressor of cellulase expression. Cre1 mediates carbon catabolite repression (CCR), a mechanism by which the fungus uses the most energetically favorable carbon source, repressing the expression of genes required for metabolization of alternative carbon sources (15–17). As eukaryotes, fungi are also subject to chromatin-mediated regulation of gene expression, and TFs need to remodel chromatin to regulate the expression of their target genes. Both Xyr1 and Cre1 are implicated in changes in the chromatin status of cellulase genes (18–20), indicating that the modulation of chromatin structure is a key step in cellulase regulation. Despite that, some studies have shown that there are mechanisms of cellulase regulation independent of Xyr1 and Cre1 (21–23). Therefore, identifying new TFs that regulate cellulase production may shed light on new and different regulatory mechanisms of cellulase expression in *T. reesei*.

Sexual development in *T. reesei* was discovered almost two decades ago. Since successful mating has been achieved, the mechanism and determinants of this process were investigated to understand *T. reesei* physiology and for industrial strain improvement (24, 25). *T. reesei* is heterothallic and presents the mating type *mat1-2*. Therefore, it needs a partner of the opposite mating type to cross. Further studies showed that *T. reesei* strain QM6a and all its derivatives (QM9414, TU6, and the mutants constructed in these backgrounds) are all female sterile due to a defect in the scaffolding protein Ham5 (24, 26).

To identify novel potential regulators of cellulase expression, we analyzed transcriptomics data from *T. reesei* grown in different carbon sources. This analysis led us to a TF encoded by the gene TRIREDRAFT_75672. Phylogenetic analysis indicated that this gene is a homolog of NsdC (*Never in sexual development*) from *Aspergilli*. This transcription factor was first identified in a screening of *Aspergillus nidulans* mutants that failed to develop cleistothecia, the fruiting body structure in *Aspergillus* (27). Further characterization showed that NsdC is a positive regulator of sexual development in *A. nidulans*. Also, *nsdC* deletion resulted in retarded growth in different carbon sources and caused an earlier development of asexual spores on solid media and produced conidiophores and viable conidia under submerged culture, showing that NsdC is a repressor of asexual development (27). In the aflatoxin-producing fungus *Aspergillus flavus*, NsdC is also important for sexual development and a repressor of conidiation in liquid culture. However, deletion of *nsdC* in *A. flavus* reduces conidiation in solid media. Furthermore, this study showed that NsdC plays a role in regulating secondary metabolite production, since the Δ*nsdC* strain failed to produce aflatoxin and aflatrem (28, 29). More recently, novel biological functions of NsdC were revealed in the opportunistic human fungal pathogen *Aspergillus fumigatus*. In *A. fumigatus*, NsdC is also important for sexual reproduction and repression of conidiation in liquid media, as reported for other fungi. Besides that, this study showed that NsdC is involved in calcium tolerance, cell wall remodeling, affecting cell wall composition and organization, and virulence, among other important biological processes (30). Our results showed that, in *T. reesei*, the NsdC homolog is also involved in vegetative growth, stress response, and calcium metabolism. In addition, this TF acts mainly as a repressor for both cellulase and cell wall biosynthesis gene expression. Our study showed that Nsd3 regulates several physiological processes and provides novel insights into the regulatory system of cellulases in *T. reesei*.

## RESULTS

### TRIREDRAFT_75672 encodes a zinc finger C_2_H_2_ transcription factor homolog of NsdC from *Aspergilli*

To identify novel transcription factors that regulate cellulase production in *T. reesei*, we analyzed transcriptomics data from *T. reesei* strains QM9414, Δ*xyr1,* and Δ*cre1* grown in cellulose, sophorose, and glucose as sole carbon sources (7, 21, 22, 31) and searched for putative *cis-*regulatory elements enriched in the promoters of genes differentially expressed under those conditions (Fig. S1). Five putative motifs were identified and compared to the database of *cis*-regulatory elements characterized in *S. cerevisiae* (Fig. S2). Among them, one motif was associated with the transcription factor Mot3p, which is of interest due to its involvement in hypoxia and osmotic stress in *S. cerevisiae* (32). Next, a BLASTp search using the Mot3p sequence was performed against the *T. reesei* genome, and four Mot3p putative homologs were identified, encoded by the genes TRIREDRAFT_58897, TRIREDRAFT_75672, TRIREDRAFT_112342, and TRIREDRAFT_36453 (identity ranging from 40 to 43%). Expression analysis of these genes in transcriptomics data from *T. reesei* strain QM6a cultivated on sugarcane bagasse indicated that only the gene TRIREDRAFT_75672 was differentially expressed, presenting a log_2_ fold change (log_2_FC) of 1.39 (adjusted *P-*value 0.001832774) (Table S1). Therefore, we focused on characterizing TRIREDRAFT_75672.

Interestingly, we were not able to identify any apparent TRIREDRAFT_75672 homolog in the *S. cerevisiae* genome using BLASTp. Phylogenetic analysis showed that Mot3p and TRIREDRAFT_75672 are phylogenetically distant (Fig. S3A). Among the four putative Mot3p homologs identified, the most phylogenetically close to Mot3p is TRIREDRAFT_58897 (Fig. S3A). Besides that, TRIREDRAFT_75672 possesses putative homologs in other filamentous fungi and is, in fact, a homolog of the transcription factor NsdC from *A. nidulans* (identity 63.4%, e-value 9.43E-0.56), *A. fumigatus* (identity 50%, e-value 7.41E-0.56), and *A. flavus* (identity 48.1%, e-value 8.60E-059) (Fig. S3B). In the cladogram, TRIREDRAFT_75672 is grouped with its homologs from *T. reesei* close relatives and is distant from its homologs in *Aspergilli* (Fig. S3A). NsdC is a Cys_2_His_2_ transcription factor, and structural analysis by SMART showed that the *T. reesei* protein possesses the three zinc fingers present in its homologs (Fig. S3B). Sequence alignment showed that the Nsd3 DNA-binding domain is highly conserved, with two C_2_H_2_ fingers with the configuration C-X_2_-C-X_12_-H-X_3_-H and a third C_2_HC-type zinc finger, present in its ortholog NsdC from *A. nidulans* (Fig. S3C) (27). Accordingly, the TRIREDRAFT_75672 gene was named *nsd3*.

### Nsd3 is required for normal growth and conidiation but is dispensable for sexual reproduction in *T. reesei*

**Fig 1:**
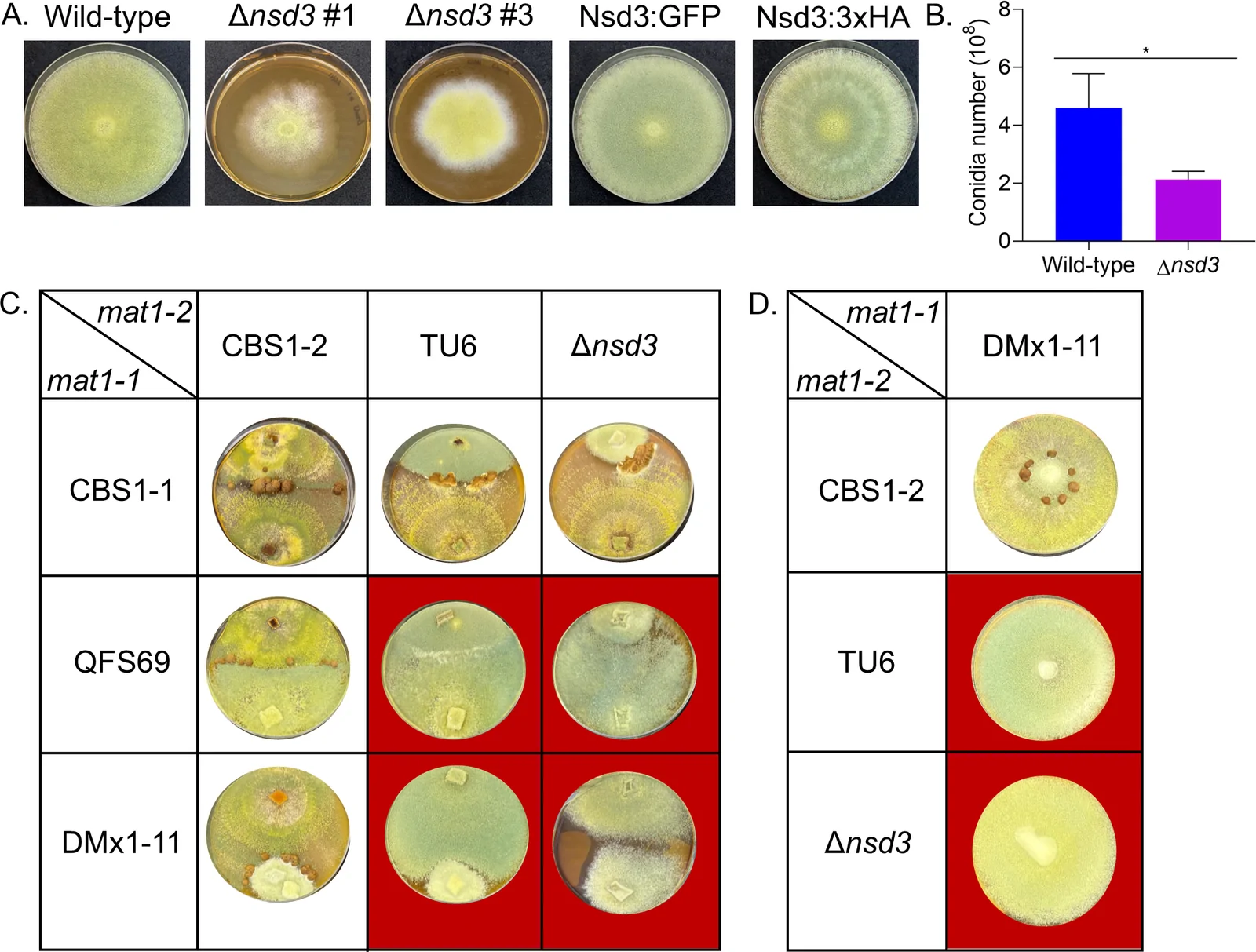
Nsd3 regulates asexual but not sexual reproduction in *T. reesei*. (**A**) The WT, Δ*nsd3*, Nsd3:GFP, and Nsd3:3×HA strains were inoculated in MEX medium and grown at 30°C. Photographs were taken after 5 days of growth. (**B**) Number of conidia of the WT and Δ*nsd3* strains. Conidia were collected after 8 days of growth in MEX medium and quantified using a Neubauer chamber. Results are the means of three biological replicates for each strain, and error bars indicate standard deviation. Asterisks indicate a significant difference (*P*-value < 0.05, *t*-test with Welch’s correction). (**C and D**) Sexual crosses between strains of opposite mating types. Strains *mat1-2* are at the top of the plate, while strains *mat1-1* are at the bottom (**C**), or an equal amount of conidia from both mating partners was inoculated in the same spot on the plate (**D**). Crosses were set in MEX medium for 14 days at 22°C under 12 h light/12 h dark to allow crossing, and then, the photos were taken. The red background indicates the absence of fruiting bodies.

To better understand the role of Nsd3 in *T. reesei* physiology, we applied the CRISPR-Cas9 system to generate a deletion mutant in the TU6 background, here referred to as the wild-type (WT) strain (Fig. S4). TU6 is a uridine auxotroph strain derived from QM9414 (33). Deletion of *nsd3* in *T. reesei* severely affected the growth on solid malt extract (MEX) medium in two independent transformants analyzed (Fig. 1A). For further characterization, the Δ*nsd3* #3 transformant was used in most experiments, hereafter referred to simply as the Δ*nsd3* strain. A significant reduction (about 2-fold) in the number of conidia produced on solid MEX was observed in the Δ*nsd3* strain (Fig. 1B). Reintroduction of the *nsd3* gene, fused to GFP (green fluorescent protein) or 3×HA (hemagglutinin) at its C-terminal region (Fig. S5), in the Δ*nsd3* strain restored the WT phenotype (Fig. 1A). These results indicate that Nsd3 is important for promoting hyphal vegetative growth and concomitant conidiation on solid media.

In *Aspergilli*, NsdC regulates sexual reproduction (27, 29, 30). Therefore, we investigated whether this role is conserved in *T. reesei*. To study the contribution of Nsd3 to *T. reesei* sexual reproduction, sexual crosses were performed using the CBS999.97 *mat1-1* (CBS1-1) strain as the sexual partner of TU6 (WT) and Δ*nsd3* strains. A cross between CBS1-1 and the CBS999.97 *mat1-2* (CBS1-2) strain was performed as a positive control. Both CBS1-1 and CBS1-2 strains are fully fertile (male and female) (24). Crosses were also performed using the QFS69 strain, which has the mating type *mat1-1* and is female sterile (34), as an additional control.

Crosses between the CBS1-1 and CBS1-2, QFS69 and CBS1-2, and CBS1-1 and TU6 strains formed fruiting bodies (Fig. 1C), as expected. The cross between CSB1-1 and Δ*nsd3* also formed fruiting bodies (Fig. 1C), indicating that Nsd3 is not essential for sexual development. Crosses between QFS69 and TU6 or Δ*nsd3* did not form fruiting bodies (Fig. 1C), consistent with all these strains being female sterile.

As *T. reesei* is a heterothallic fungus, the impact of *nsd3* absence on both mating types was investigated. We screened the offspring from the cross between CBS1-1 and Δ*nsd3* and recovered one strain, DMx1-11, that proved to be *mat1-1* and knockout for *nsd3* (Fig. S6A and B). The DMx1-11 strain also showed growth defects, consistent with the phenotype observed in the Δ*nsd3* strain in the TU6 background (Fig. S6C). Crosses between DMx1-11 and CBS1-2 formed fruiting bodies (Fig. 1C), indicating that Nsd3 is also not essential for sexual reproduction in the mating type *mat1-1*. Crosses between DMx1-11 and TU6 or Δ*nsd3* did not form fruiting bodies, indicating that DMx1-11 is possibly female sterile. A small clearing zone is observed in the plates of these crosses, indicating that the deletion of *nsd3* in this mating type may be affecting the production of secondary metabolites and, therefore, the chemical communication between the strains. To overcome the growth defects presented by the *nsd3* knockout strains, we mixed an equal amount of conidia of both mating partners in the same spot on the plate. Again, crosses between CBS1-2 and DMx1-11 formed fruiting bodies, but crosses between DMx1-11 and TU6 or Δ*nsd3* did not (Fig. 1D). These results show that Nsd3 is not essential for sexual development in *T. reesei*, even when absent in both sexual partners.

### Nsd3 is involved in the stress response and calcium metabolism

**Fig 2:**
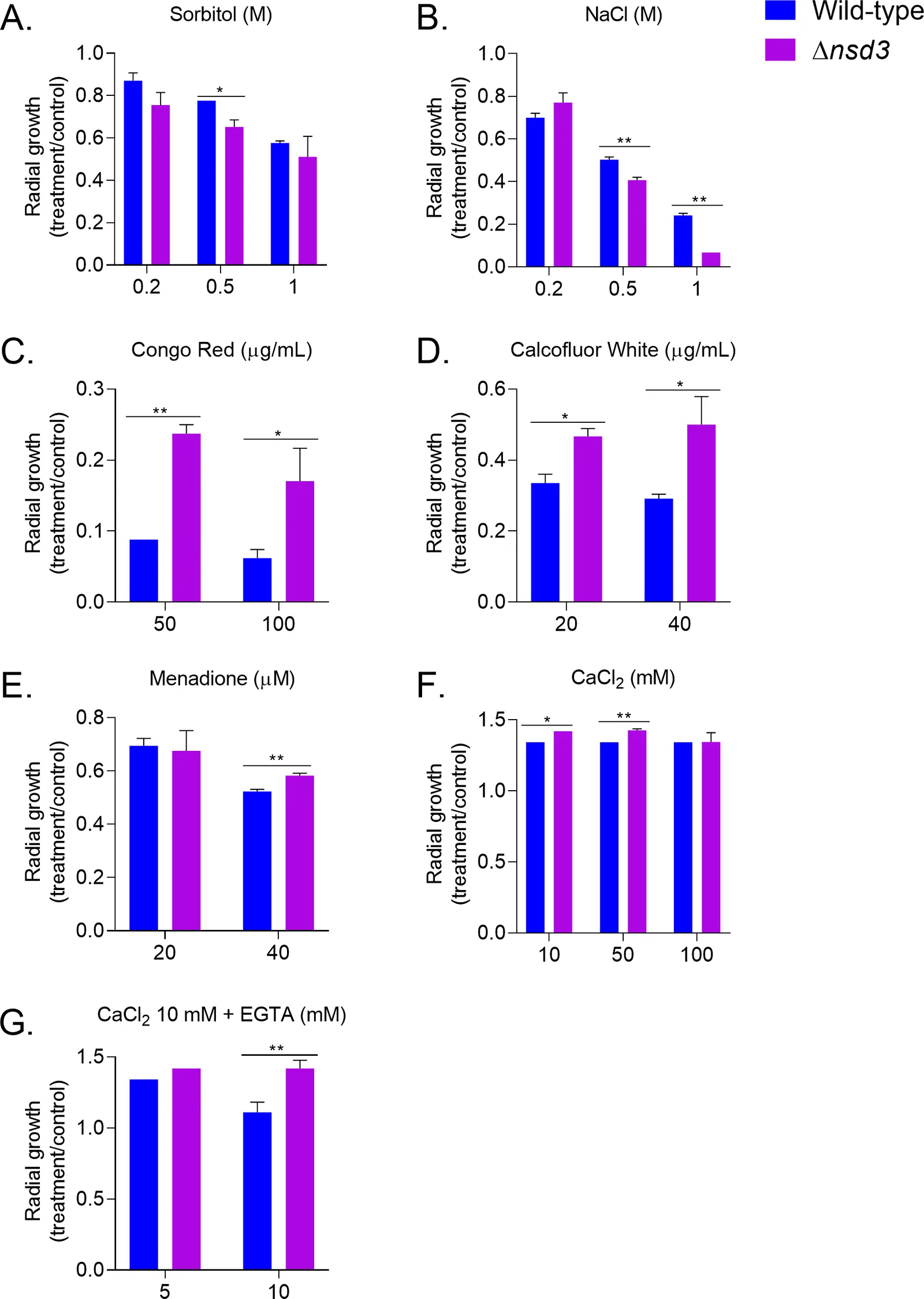
Nsd3 regulates the response to different stresses. (**A–G**) WT and Δ*nsd3* strains were cultured for 5 days on solid GMM in the presence of different concentrations of sorbitol (**A**), NaCl (**B**), Congo red (**C**), Calcofluor White (**D**), menadione (**E**), CaCl_2_ (**F**), and EGTA (**G**). Results are expressed as radial growth of the treatment/radial growth of the control (without any stressor). For the analysis of growth in the presence of EGTA, the control condition was growth in the presence of 10 mM CaCl_2_. Results are the means of three biological replicates for each strain, and error bars indicate standard deviation. Asterisks indicate significant differences (*P ≤ 0.05, **P ≤ 0.01, ***P ≤ 0.001, ****P ≤ 0.0001, *t*-test with Welch’s correction).

Our initial goal was to identify novel TFs that regulate both cellulase production and stress response in *T. reesei*. Based on this, WT and Δ*nsd3* strains were subjected to different types of stress: sorbitol and NaCl (osmotic stress), CaCl_2_ (ionic stress), EGTA (calcium chelator), Calcofluor White (CFW), and Congo red (CR) (cell wall stress), and menadione (oxidative stress) (35). The *Δnsd3* strain proved to be more sensitive than the WT to osmotic stress caused by 0.5 M sorbitol and by NaCl (Fig. 2A and B). While the WT strain grew poorly in the presence of CR, the Δ*nsd3* strain was able to tolerate up to 100 μg/mL of CR (Fig. 2C) and is also more resistant to CFW than the WT (Fig. 2D). Furthermore, the Δ*nsd3* strain is more resistant to the oxidative stress caused by menadione (Fig. 2E). These results indicate that Nsd3 is necessary for the response to osmotic stress but acts as a repressor of the expression of genes involved in the response to cell wall and oxidative stresses.

In *A. fumigatus,* NsdC is involved in calcium signaling (30). Therefore, the tolerance of the Δ*nsd3* strain to CaCl_2_ was investigated. The addition of CaCl_2_ improves growth in both WT and Δ*nsd3* strains (Fig. 2F), as previously reported (36). However, at concentrations of 10 and 50 mM, calcium positively affects the growth of the Δ*nsd3* strain more than that of the WT. Furthermore, the Δ*nsd3* strain is more resistant than the WT to the calcium chelator EGTA at 10 mM (Fig. 2G). These results suggest that, as in *A. fumigatus*, Nsd3 plays a role in calcium metabolism and signaling in *T. reesei*.

### Nsd3 is a repressor of cellulase expression

**Fig 3:**
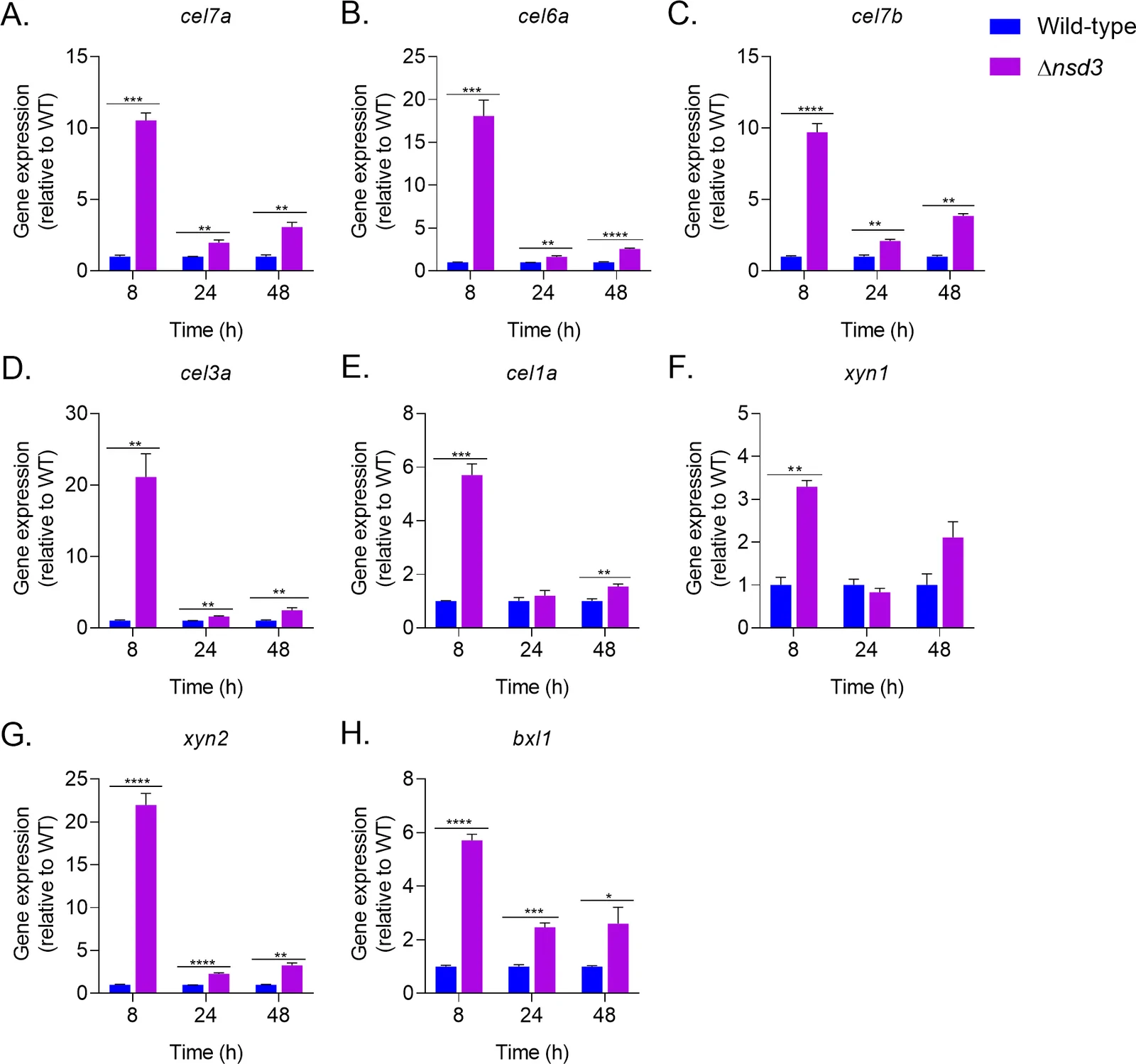
Nsd3 regulates holocellulase expression in *T. reesei*. (**A–H**) Gene expression of the main cellulases and hemicellulases produced by *T. reesei*. WT and Δ*nsd3* strains were cultured on cellulose as the sole carbon source for 8, 24, and 48 h for RNA extraction, after pregrowing on glycerol for 24 h. Gene expression was analyzed by RT-qPCR and calculated based on the 2^−ΔCt^ method, using the actin gene as an endogenous control. Results are represented as relative expression in relation to the WT strain (set as 1) at the respective time point. Results are the means of three biological replicates for each strain, and error bars indicate standard deviation. Asterisks indicate significant differences (*P ≤ 0.05, **P ≤ 0.01, ***P ≤ 0.001, ****P ≤ 0.0001, *t*-test with a Holm-Sidak post-hoc test).

Next, we investigated the role of Nsd3 in cellulase production. WT and Δ*nsd3* strains were exposed for 8, 24, and 48 h to cellulose to induce cellulase production, and the expression of the main cellulases and hemicellulases produced by *T. reesei* was analyzed by RT-qPCR. As shown in Fig. 3, deletion of *nsd3* positively impacts the expression of holocellulases in response to cellulose. The expression levels of *cel7a*, the most important cellulase produced by *T. reesei*, are 10-fold higher in the Δ*nsd3* strain after 8 h of culture on cellulose (Fig. 3A). This difference in *cel7a* expression between the strains decreases with the culture time but remains significantly higher in the Δ*nsd3* strain. A similar profile was observed for the endoglucanase *cel7b* (Fig. 3C). The *cel6a*, *cel3a*, and *xyn2* genes were the most impacted by the *nsd3* deletion, being upregulated in the knockout strain (about 20-fold after 8 h of culture on cellulose) (Fig. 3B, D and G). Although they were also upregulated in the Δ*nsd3* strain at 8 h, the expression of *cel1a* and *xyn1* is at the same level as the WT after 24 h of culture (Fig. 3E and F). *bxl1* is significantly more expressed in Δ*nsd3* at all time points analyzed (Fig. 3H). These results show that Nsd3 represses the expression of holocellulases in *T. reesei* and that its deletion causes a fast and robust activation of the expression of holocellulolytic genes after induction with plant biomass.

**Fig 4:**
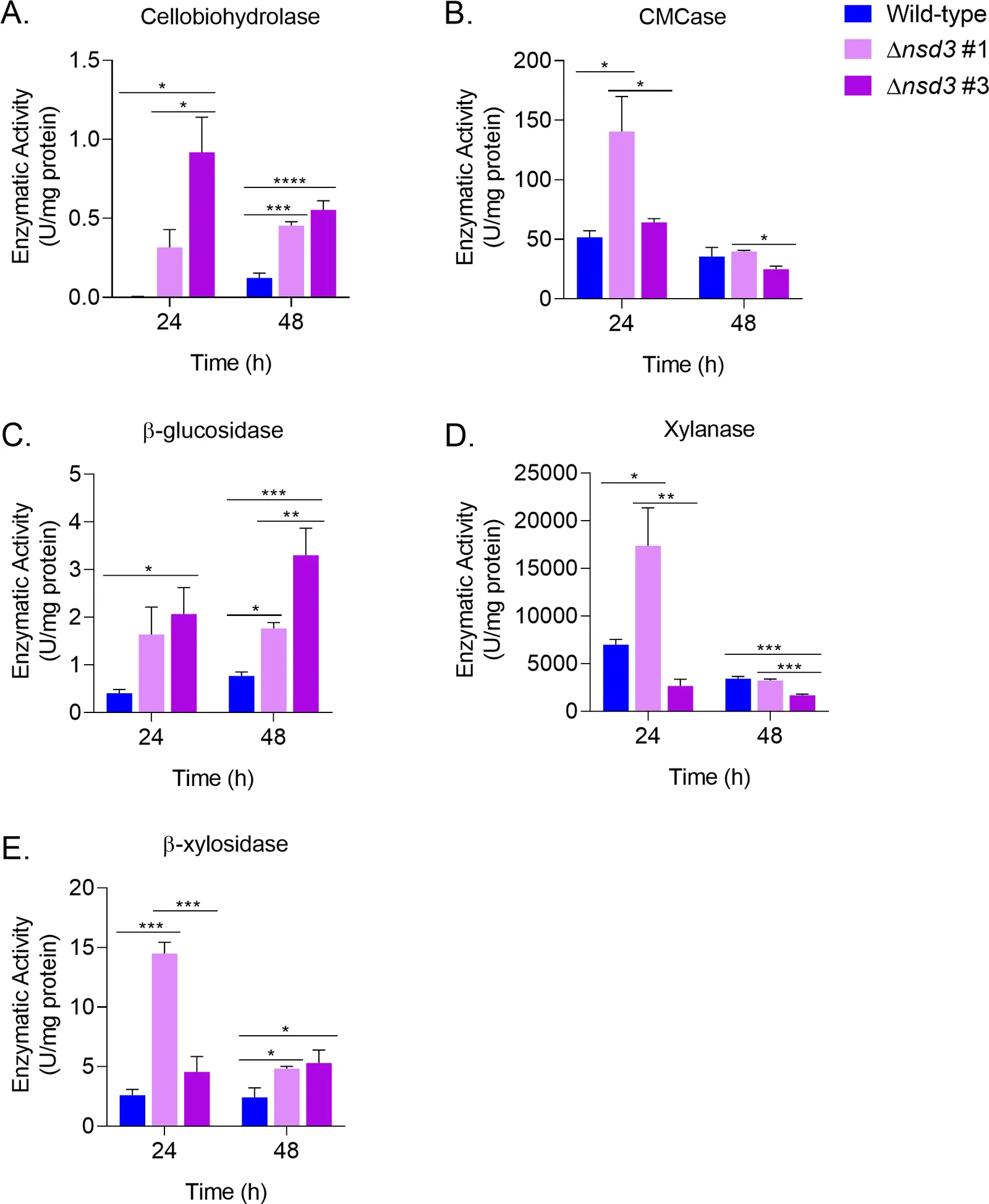
Nsd3 affects the *T. reesei* enzymatic production. Cellulolytic and hemicellulolytic activities of WT and Δ*nsd3* strains after growth on cellulose (after pregrowing on glycerol for 24 h). Culture supernatants were collected at the indicated time and used to quantify cellobiohydrolase (**A**), CMCase (**B**), β-glucosidase (**C**), xylanase (**D**), and β-xylosidase (**E**). Results are the means of three biological replicates for each strain, and error bars indicate standard deviation. Asterisks indicate significant differences (*P ≤ 0.05, **P ≤ 0.01, ***P ≤ 0.001, ****P ≤ 0.0001, one-way ANOVA with a Tukey post-hoc test).

Cellulase and hemicellulase activities were tested in the WT and Δ*nsd3* strains after growing for 24 and 48 h in cellulose, after previous growth in glycerol for 24 h. The Δ*nsd3* #1 transformant was included in this assay to confirm the phenotype. Deletion of *nsd3* caused a significant increase in the specific activities of cellobiohydrolase (Fig. 4A), β-glucosidase (Fig. 4C), and β-xylosidase (Fig. 4E). Although a significant increase in the specific activities of CMCase (Fig. 4B) and xylanase (Fig. 4D) was observed at 24 h for the Δ*nsd3* #1 strain, after 48 h of culture, the activities returned to the same levels as the WT, being significantly lower in the Δ*nsd3* #3 strain. We hypothesize that these differences between the two Δ*nsd3* transformants may be due to variability in the fungal secretome during cultivation. Furthermore, the DMx1-11 also showed higher levels of cellulase activity relative to the WT strain (Fig. S6D).

Since deletion of *nsd3* has been shown to increase cellulase production, we asked whether it participates in the CCR by growing the WT and Δ*nsd3* strains on cellobiose with increasing concentrations of 2-deoxyglucose (2-DG), a glucose analog that cannot be metabolized. Strains with defects in CCR are insensitive to 2-DG (37). The Δ*cre1* strain was included in this assay as a control. The results show that both WT and Δ*nsd3* strains are sensitive to 2-DG, since no growth was observed after 3 days of cultivation, except for Δ*cre1* (Fig. S7), indicating that Nsd3 does not contribute to CCR in *T. reesei*. Altogether, these results show that Nsd3 negatively regulates holocellulase production in *T. reesei*, affecting the expression and secretion of these enzymes through a mechanism independent of CCR.

### Transcriptional profile of the Δ*nsd3* strain

Our results show that Nsd3 regulates diverse physiological processes in *T. reesei*. Therefore, we investigated the impact of the deletion of *nsd3* on *T. reesei* global gene expression through RNA sequencing (RNA-Seq). WT and Δ*nsd3* strains were cultivated on glycerol, glucose (both for 24 h), or cellulose for 8 and 48 h (after pregrowing on glycerol for 24 h) before harvesting the cells for RNA-Seq (Fig. S8A). Differentially expressed genes (DEGs) were defined as those with a minimum of 2-fold change in gene expression (|log_2_FC| ≥ 1, adjusted *P*-value < 0.05).

On glycerol, 1,120 genes are differentially expressed (475 upregulated and 645 downregulated). Functional categorization (FunCat) analysis showed that upregulated genes are involved in the transport of different molecules (amines and polyamines, siderophores, carbohydrates and C-compounds, ions, drugs, and toxins), detoxification, proton homeostasis, and secondary metabolism (Fig. S8D). Genes involved in the degradation of extracellular polysaccharides, protein degradation, cell differentiation, carbon and polysaccharide metabolism, sesquiterpene metabolism, and secondary metabolism are enriched among the downregulated genes (Fig. S8D). Among the most upregulated genes in the Δ*nsd3* strain, we found the sugar transporter Lac1 (TRIREDRAFT_56684), which is important for cellulase production in *T. reesei* (38).

**Fig 5:**
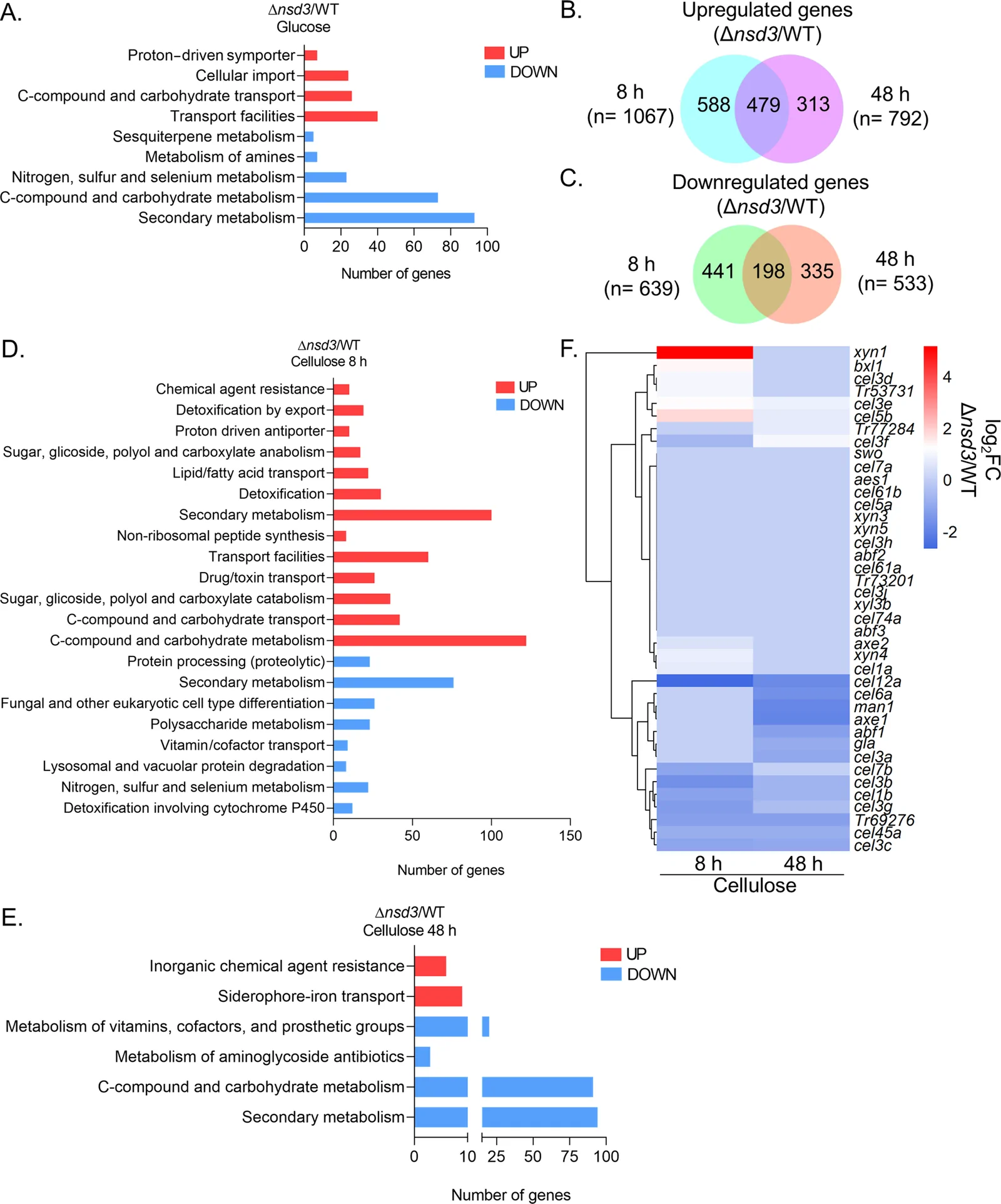
Transcriptional profile of the Δ*nsd3* strain. (**A**) Functional enrichment analysis using FunCat terms for differentially expressed genes in the Δ*nsd3* strain relative to the WT on glucose. An adjusted *P*-value < 0.05 was used to filter the terms. (**B and C**) Venn diagrams comparing upregulated (**B**) and downregulated (**C**) genes in the Δ*nsd3* strain relative to the WT strain after cultivation for 8 and 48 h on cellulose. (**D and E**) Functional enrichment analysis using FunCat terms for differentially expressed genes in the Δ*nsd3* strain relative to the WT strain after cultivation for 8 h (**D**) and 48 h (**E**) on cellulose. An adjusted *P*-value < 0.05 was used to filter the terms. (**F**) Heatmap showing gene expression (in log_2_FC) of *T. reesei* main holocellulases at 8 and 48 h on cellulose.

On glucose, 1,154 genes are differentially expressed (590 upregulated and 564 downregulated). FunCat analysis showed that the upregulated genes are involved in the transport of molecules such as carbohydrates and ions, while the downregulated genes showed enrichment for genes involved in the metabolism of sesquiterpenes, amines, nitrogen, sulfur, and selenium, as well as carbohydrate and C-compound metabolism and secondary metabolism (Fig. 5A). Among the most upregulated genes in the Δ*nsd3* strain on glucose, we found only one encoding a holocellulase: TRIREDRAFT_54219, which encodes a CE5 acetyl xylan esterase.

On cellulose, 1,067 and 792 genes are upregulated in the Δ*nsd3* strain at 8 and 48 h, respectively, and only 479 genes are upregulated in both conditions (Fig. 5B). FunCat analysis shows that the genes upregulated at 8 h are involved in processes such as carbohydrate and C-compound metabolism and transport, lipid and fatty acid transport, detoxification, and secondary metabolism (Fig. 5D), while the genes upregulated at 48 h are involved in resistance to inorganic chemical agents and in iron and siderophore transport (Fig. 5E). At 8 h, 639 genes are downregulated in the Δ*nsd3* strain compared to the WT, whereas 533 are downregulated at 48 h, with 198 shared between the conditions (Fig. 5C). At 8 h, these downregulated genes are enriched for protein homeostasis (proteolysis and degradation in vacuoles and lysosomes), cell differentiation, polysaccharide, nitrogen, sulfur, and selenium metabolism, vitamin/cofactor transport, detoxification involving cytochrome P450, and secondary metabolism (Fig. 5D), whereas the downregulated genes at 48 h are involved in vitamin and cofactor metabolism, aminoglycoside antibiotics, carbohydrate and C-compound metabolism, and secondary metabolism (Fig. 5E). The RNA-Seq results show that Nsd3 is required for proper adaptation to different carbon sources, regulating a distinct set of genes in each. Furthermore, Nsd3 acts primarily as a repressor of gene expression during the induction of cellulase production, modulating genes involved in biomass degradation, transport and metabolism of biomass-derived carbohydrates, and secondary metabolism.

Next, we profiled the expression of *T. reesei* main holocellulases in the transcriptomes of the Δ*nsd3* strain on cellulose. In agreement with RT-qPCR data, the deletion of *nsd3* affects holocellulase expression primarily in the early cellulose cultivation time (8 h), where a greater number of genes are upregulated, especially *xyn1* and *cel5b*. At the same time, some genes are downregulated, particularly some endoglucanases and β-glucosidases genes (Fig. 5F). At 48 h, Nsd3 has less influence on the expression of most holocellulolytic genes, and only a few genes are downregulated. One group of genes was not modulated by Nsd3 at any of the analyzed time points (Fig. 5F). These results indicate that Nsd3 influences the expression of different holocellulases, repressing their expression in the early stages of lignocellulosic biomass degradation by *T. reesei*.

### Chromatin accessibility profile in the Δ*nsd3* strain

**Fig 6:**
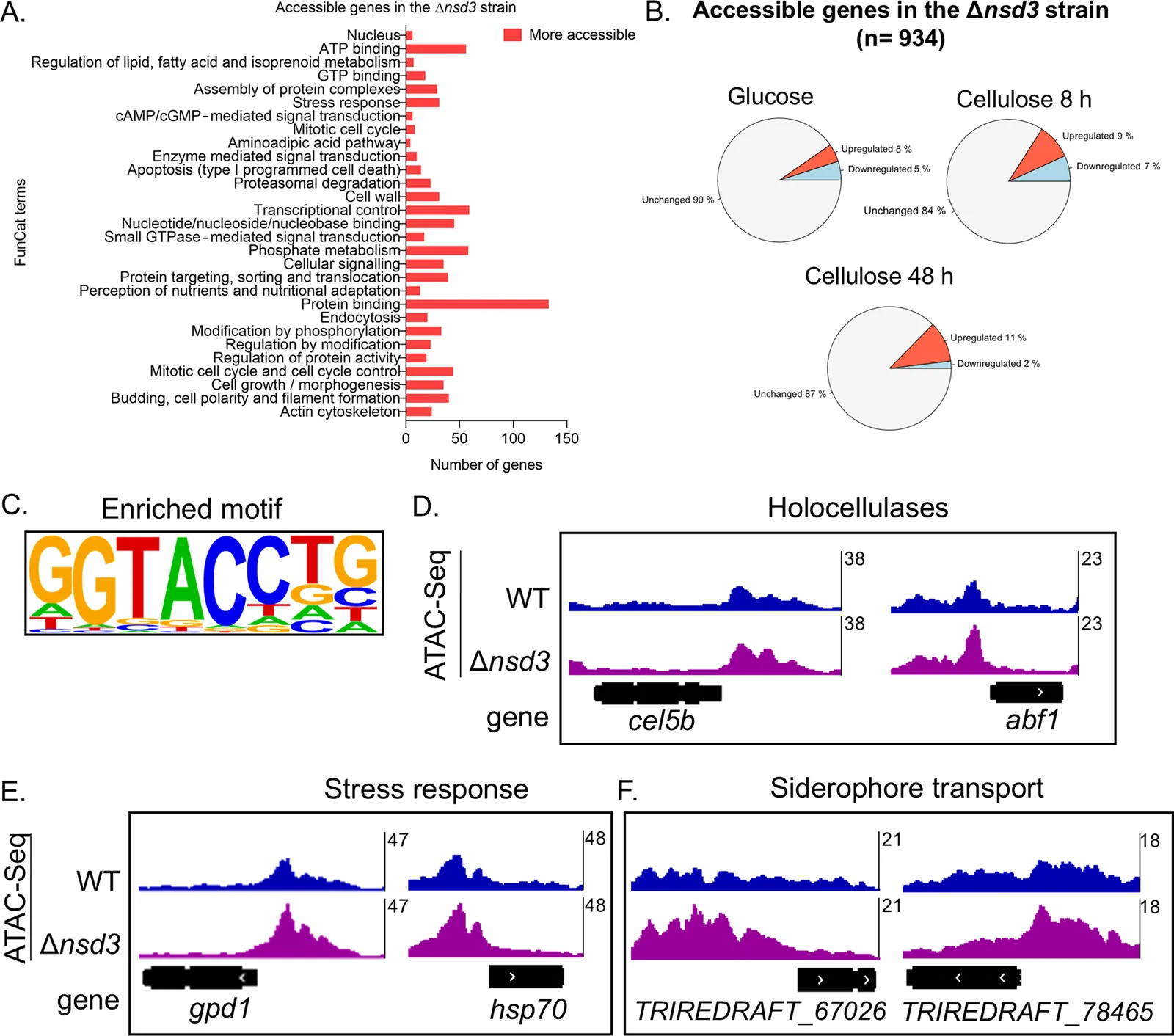
Characterization of regions with accessible chromatin in the Δ*nsd3* strain. (**A**) Functional enrichment analysis using FunCat terms for the more accessible genes in the Δ*nsd3* strain compared to the WT strain on glucose. An adjusted *P*-value < 0.05 was used to filter the terms. (**B**) Analysis of the expression profile of accessible genes in Δ*nsd3* in RNA-Seq data from glucose and cellulose (8 and 48 h). (**C**) Enriched binding motif in accessible chromatin regions in the Δ*nsd3* strain. (**D–F**) Genome Browser screenshots showing the ATAC-Seq enrichment signal in the WT (blue) and Δ*nsd3* (purple) strains in the promoters of genes encoding holocellulases (**D**), genes involved in the stress response (**E**), and genes encoding siderophore transporters (**F**).

To obtain more information about the role of Nsd3 in the physiology of *T. reesei*, the assay for transposase-accessible chromatin sequencing (ATAC-Seq) was performed to identify genome-wide regions with accessible chromatin in the Δ*nsd3* strain relative to the WT. These experiments were performed only on glucose, being not successful on cellulose. To assess ATAC-Seq efficiency, we examined the genes encoding the glycolytic enzyme hexokinase (*hxk*, TRIREDRAFT_80231) and *cel7a* as controls. As expected, these genes exhibited accessible and inaccessible promoters, respectively, in both WT and Δ*nsd3* strains (Fig. S10A). After data analysis, regions with accessible chromatin in the Δ*nsd3* strain were associated with 934 genes. These data were analyzed qualitatively: genes considered more accessible in the Δ*nsd3* are those identified exclusively in this strain, whereas those identified only in the WT strain were considered less accessible. FunCat analysis of the more accessible genes in the Δ*nsd3* strain showed that they are involved in several biological processes, notably protein binding, ATP and GTP binding, phosphate metabolism, protein homeostasis, cell signaling, growth and cell cycle, cell wall, nutrient sensing, and stress response (Fig. 6A). The less accessible genes did not show significant enrichment for any biological process (adjusted *P*-value > 0.05), but manual inspection of the genes indicates that they are involved in processes, such as protein binding, cell division, and vesicle formation.

Next, the expression profile of genes with accessible chromatin in the Δ*nsd3* strain was analyzed using RNA-Seq data. On glucose, only 10% of the genes had their expression modulated in the mutant strain, with 5% upregulated and 5% downregulated (Fig. 5B), indicating that, although the promoters of these genes are more accessible, transcription activators are still needed to increase their expression. On cellulose, the number of upregulated genes increases slightly, reaching 11% at 48 h (Fig. 5B). *De novo* motif discovery analysis by HOMER identified a set of DNA motifs enriched in the accessible regions in the Δ*nsd3* strain (Fig. S10B). The most significant motif presents the consensus sequence 5′-GGTACCNB-3′ (*P*-value 1e-55) (Fig. 5C).

The more accessible genes in the Δ*nsd3* strain were manually inspected. Only two genes encoding holocellulases were identified: the endoglucanase *cel5b* and the arabinofuranosidase *abf1* (Fig. 5D). This can be explained by the fact that the chromatin is extremely condensed in the genomic regions of the genes encoding holocellulases under catabolic repression conditions (18, 39). Different genes involved in the response to several stresses, whose promoters are more accessible in the Δ*nsd3* strain, were also found, such as *gpd1*, which encodes a glycerol-3-phosphate dehydrogenase that participates in the osmotic stress response, and the chaperone *hsp70*, which acts in protein folding and the stress response (Fig. 5E). Furthermore, two genes encoding siderophore transporters, TRIREDRAFT_67026 and TRIREDRFAT_78465, are also more accessible in the mutant strain (Fig. 5F). Genes related to siderophore transport are enriched among the upregulated genes in the Δ*nsd3* strain on glycerol and cellulose (Fig. 5E and Fig. S8D). These results show that the deletion of *nsd3* alters chromatin accessibility in promoters of several genes involved in different physiological processes in *T. reesei*, but some cell signaling mechanism is still necessary to activate their expression.

### Sugar transporters are important targets of Nsd3

**Fig 7:**
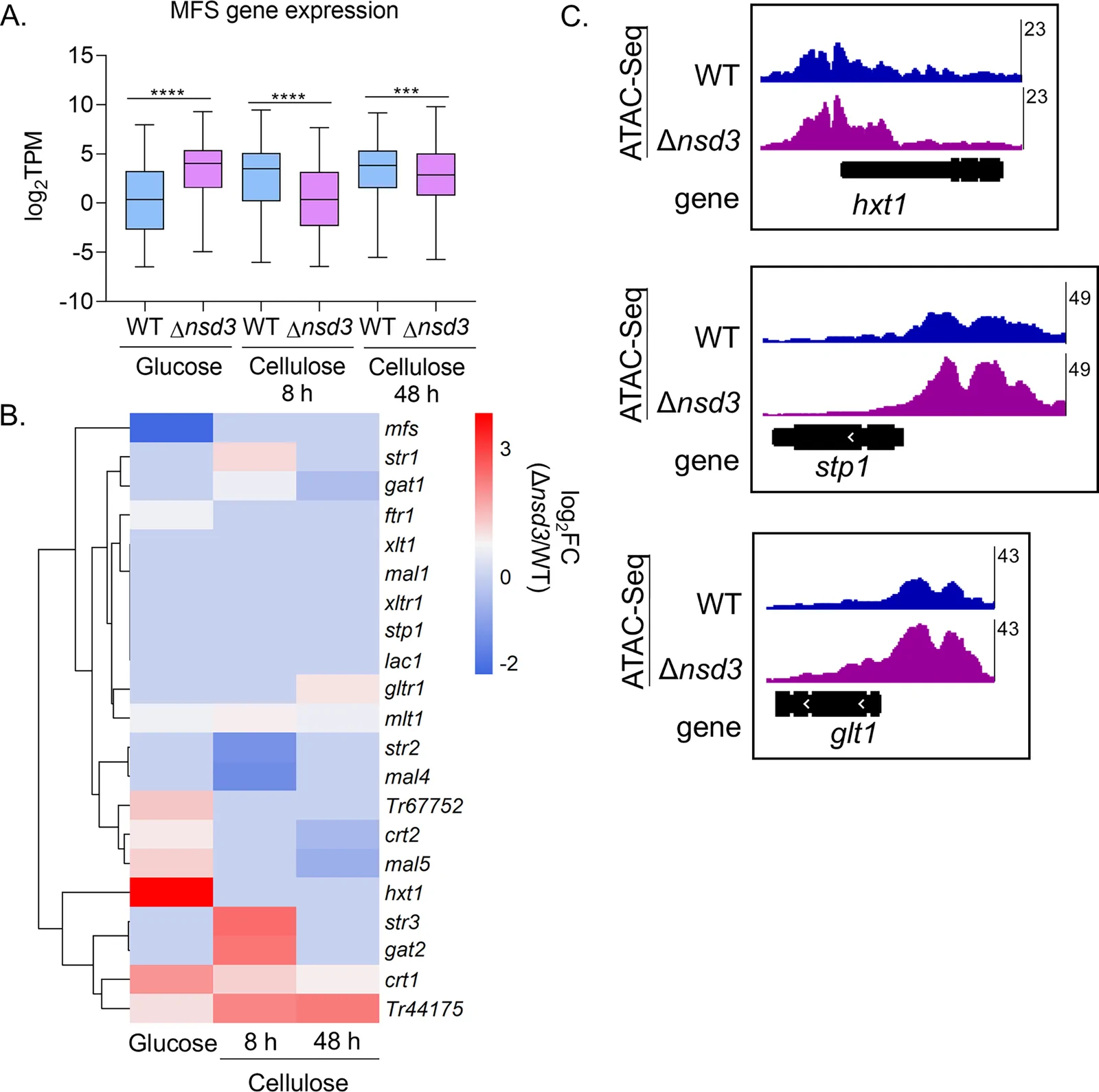
Integrative analysis of RNA-Seq and ATAC-Seq data shows that sugar transporters are important targets of Nsd3. (**A**) Box plot representing the gene expression of MFS transporters in the transcriptomes of WT and Δ*nsd3* strains in glucose and cellulose at 8 and 48 h. Values are in log_2_ transcripts per million (TPM). Asterisks indicate significant differences (***P ≤ 0.001, ****P ≤ 0.0001, Student’s *t*-test with a Holm-Sidak post-hoc test). (**B**) Heatmap showing the gene expression values of characterized sugar transporters in *T. reesei* in the analyzed transcriptomes. Values are in log_2_FC. (**C**) Genome Browser screenshots showing the ATAC-Seq enrichment signal for WT (blue) and Δ*nsd3* (purple) strains in the promoters of the characterized transporters *hxt1, stp1,* and *glt1*.

Our transcriptomics data show that genes involved in the transport of biomolecules are enriched among the upregulated genes in the Δ*nsd3* strain under all analyzed conditions. Sugar transporters play an important role in biomass degradation by *T. reesei* and can also act in the regulation of cellulase production. It is predicted that the *T. reesei* genome encodes 71 putative sugar transporters of the major facilitator superfamily (MFS), the main class of sugar transporters characterized in fungi (40). Analysis of the expression of these transporters showed that they are more expressed in the Δ*nsd3* strain than in the WT on glucose (Fig. 7A). However, on cellulose, they are less expressed in the mutant, at both time points analyzed (Fig. 7A). In this analysis, we focused on the expression data on glucose and cellulose, as they are more relevant to the context of cellulase production.

Some of these transporters have already been characterized, either by functional genomics in *T. reesei* or by heterologous expression in other systems, such as yeast. The transcriptional profile of the 21 characterized sugar transporters from *T. reesei* was analyzed in the RNA-Seq data (Fig. 7B). It is possible to observe that the expression of some of them is not affected by the deletion of *nsd3*, such as *mal1*, *lac1*, and *stp1*. In glucose, the only one that showed downregulation was the *mfs* transporter, while *Tr67752*, *crt2, mal5*, and *hxt1* were upregulated. Furthermore, the analysis of ATAC-Seq data indicates that the deletion of *nsd3* impacts the chromatin accessibility in the promoters of some genes that encode MFS transporters. Among them, the only ones already characterized are *hxt1*, *stp1,* and *glt1* (Fig. 7C). All of them have a more accessible promoter in the mutant strain. Stp1 and Glt1 constitute the dual-affinity glucose system, along with Xltr1 (10, 41). Stp1 and Xltr1 are high-affinity glucose transporters, while Glt1 is a low-affinity transporter (10, 12). Although Stp1 and Glt1 are not differentially expressed in RNA-Seq data, ATAC-Seq data suggest that Nsd3 may exert some regulation on the expression of these glucose transporters. Furthermore, Hxt1 is also a glucose transporter (42), suggesting that Nsd3 regulates the expression of important glucose transporters during growth on this carbon source.

Besides *hxt1*, the transporters *Tr67752, crt2,* and *mal5* are also upregulated in response to glucose. Tr67752 and Mal5 are cellobiose transporters, with the latter being important for cellulase production (43, 44). Crt2 plays a role in cellulase production regulation in *T. reesei,* but its transport activity and substrates have not yet been elucidated (45).

In 8 h cellulose, *mal4* and *str2* are downregulated, while the genes *str3* and *gat2* are upregulated (Fig. 7B). Mal4 is an important cellobiose transporter for cellulase production, while Str2 transports monosaccharides, such as glucose and xylose (44, 46). Str3 is also a monosaccharide transporter, possessing high affinity for glucose and mannose, while Gat2 transports uronic acid (10). The cellobiose and sophorose transporters, Crt1 (9) and Tr44175 (11), were upregulated under all analyzed conditions. These results show that Nsd3 modulates the expression of different sugar transporters, with different affinities and substrates. Specifically, the deletion of *nsd3* causes an increase in the expression of important cellobiose and sophorose transporters, molecules that activate cellulase production, which may culminate in the increased cellulase production observed in the Δ*nsd3* strain.

### Nsd3 directly binds to the promoters of key genes in the cellulolytic pathway

**Fig 8:**
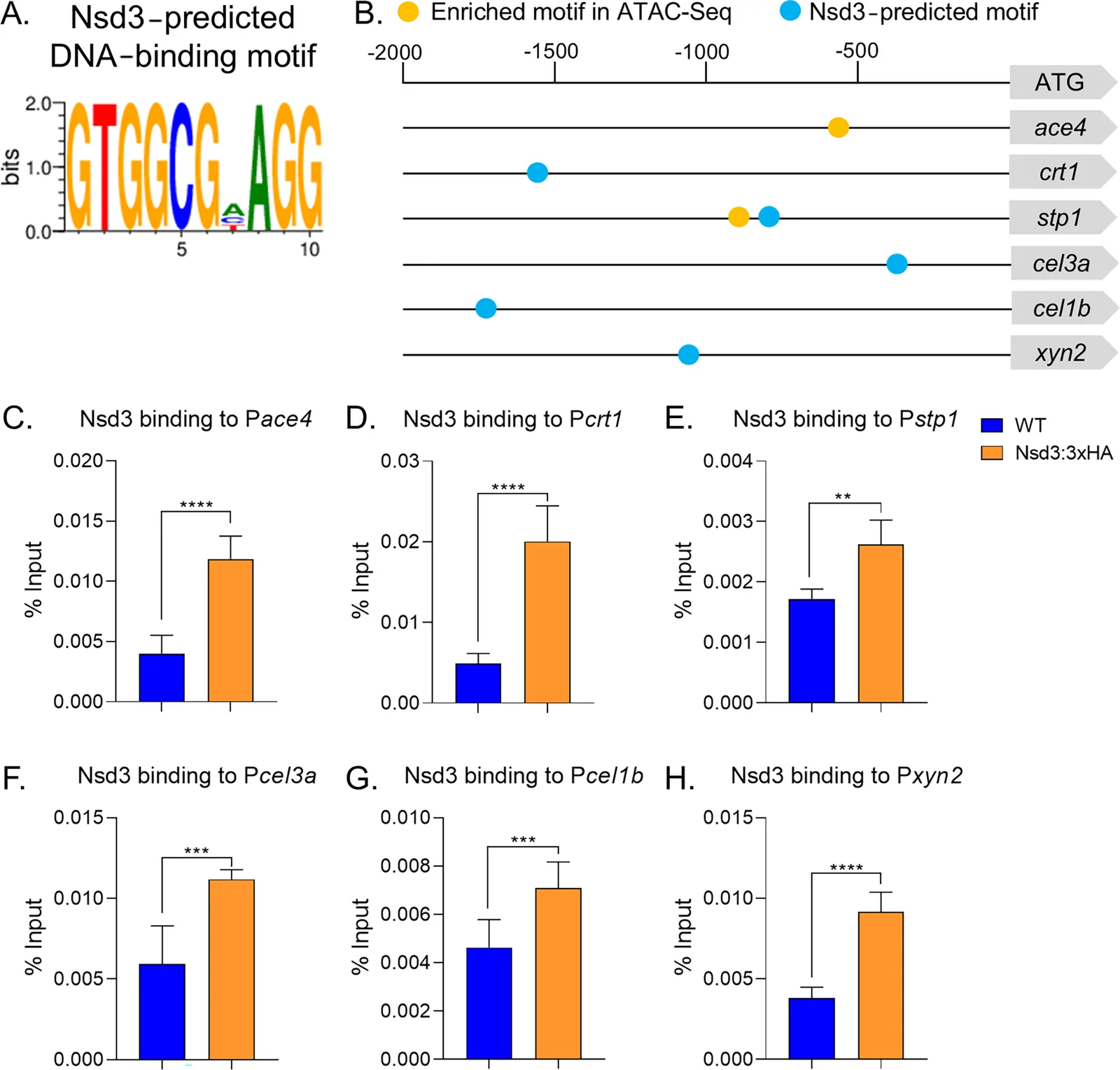
Nsd3 binds to the promoters of key genes in the cellulolytic pathway. (**A**) Nsd3 putative DNA-binding motif. (**B**) Putative binding sites for the ATAC-Seq-enriched motif (yellow) and the Nsd3-binding motif (light blue) in the promoters of the selected genes. (**C–F**) ChIP-qPCR of the *ace4* (**C**), *crt1* (**D**), *stp1* (**E**), *cel3a* (**F**), *cel1b* (**G**), and *xyn2* (**H**) promoter genes in the WT and Nsd3:3×HA strains after growth in MAM with cellulose for 6 h (pregrowing in glycerol for 24 h). Results are the means of three biological replicates for each strain, and error bars indicate standard deviation. Asterisks indicate significant differences (**P ≤ 0.01, ***P ≤ 0.001, ****P ≤ 0.0001, *t*-test with Welch’s correction).

To investigate whether Nsd3 can directly control the expression of cellulolytic genes, we performed chromatin immunoprecipitation coupled with quantitative PCR (ChIP-qPCR) in the Nsd3:3×HA and WT strains for selected genes. First, we predicted the Nsd3 putative DNA-binding motif (Fig. 8A) using the web server http://zf.princeton.edu/index.php (47). We searched for its putative binding sites in the promoters of genes involved in cellulase production using MEME (48), along with the enriched motif retrieved from the ATAC-Seq data (Fig. 6C). After this analysis, we selected the genes encoding the transcription factor *ace4*, which exhibits an accessible promoter in the Δ*nsd3* strain in the ATAC-Seq (Data Set S2), presents a putative binding site for the ATAC-Seq motif, and is upregulated in the RNA-Seq (Data Set S1); *crt1*, *cel3a*, *cel1b*, and *xyn2*, which are differentially expressed in either the RNA-Seq or RT-qPCR and present the putative Nsd3-binding sites; and *stp1*, which exhibits an accessible promoter in the Δ*nsd3* strain in the ATAC-Seq and presents putative binding sites for the ATAC-Seq and the Nsd3-binding motifs (Fig. 8B). Primer pairs targeting the identified putative Nsd3-binding regions at the promoter were used to quantify the precipitated fragments by real-time PCR. The ChIP-qPCR results of the Nsd3:3×HA strain showed significant *in vivo* binding of Nsd3 to the promoter regions of all tested genes compared to the negative (no 3×HA) control WT strain (Fig. 8C through H). Taken together, these results show that Nsd3 regulates cellulase production in *T. reesei* by directly controlling the expression of important transcription factors (e.g., Ace4), sugar transporters (e.g., Crt1 and Stp1), and holocellulases (e.g., Cel3a, Cel1b, and Xyn2).

### Nsd3 regulates cell wall organization and maintenance

**Fig 9:**
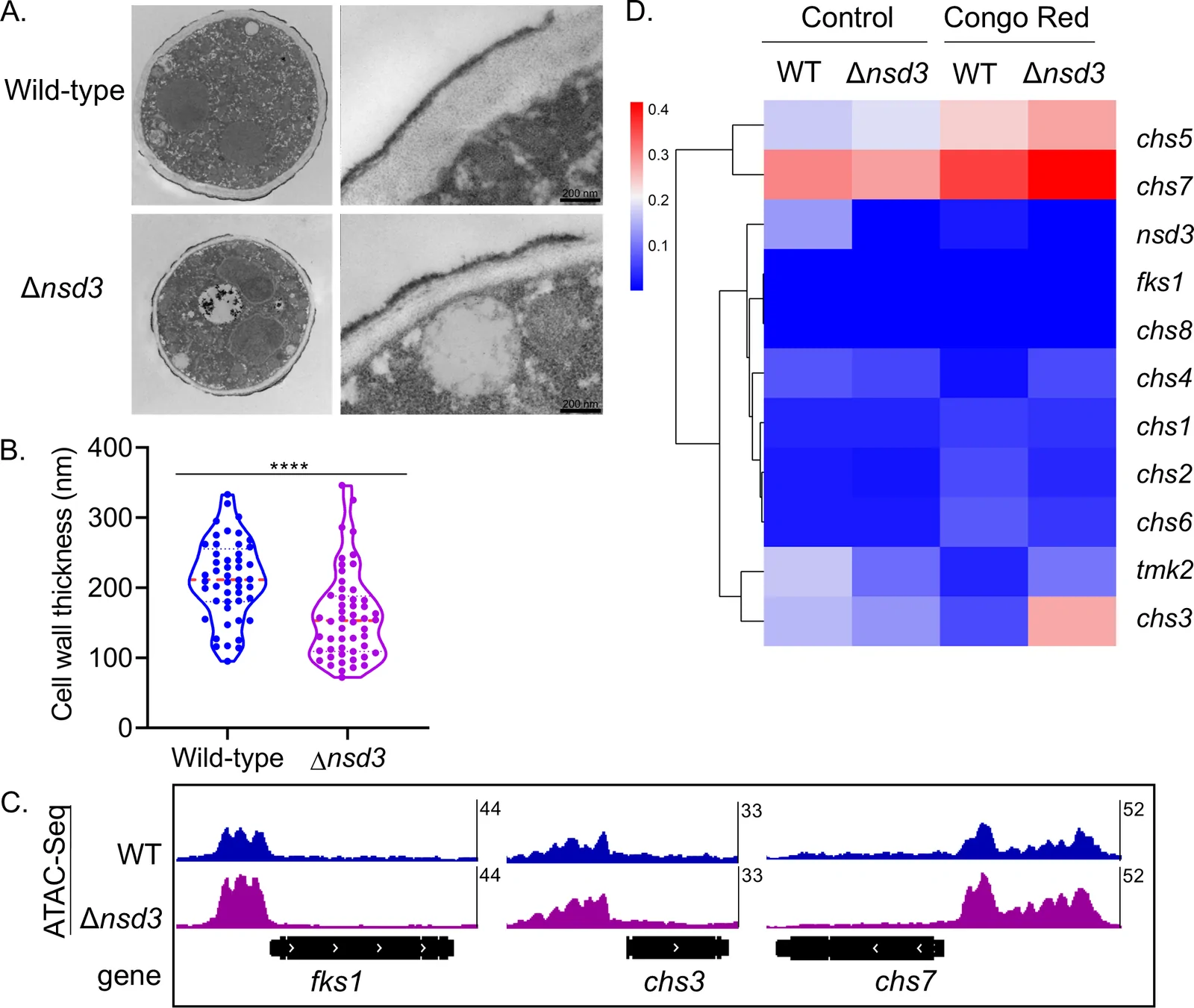
Nsd3 regulates cell wall remodeling. (**A**) Transmission electron microscopy of mycelial sections of WT and Δ*nsd3* strains after static growth in MAM with glucose for 18 h. (**B**) Violin plot showing the cell wall thickness (nanometers) of approximately 50 sections of different hyphae for each strain. Dashed lines indicate quartiles, and red lines indicate the median for each condition. Asterisks indicate significant differences (****P ≤ 0.0001) as assessed by *t*-test with Welch’s correction. (**C**) Genome Browser screenshots showing the ATAC-Seq enrichment signal for WT (blue) and Δ*nsd3* (purple) strains in promoters of genes related to cell wall biosynthesis. (**D**) Nsd3 affects the expression of genes involved in cell wall integrity. Heatmap showing the expression of genes involved in cell wall integrity after exposure to Congo red stress. WT and Δ*nsd3* strains were cultured in MAM with glucose for 24 h, and then, the cells were treated with CR 100 μg/mL for 1 h. Untreated cells were used as a control. The expression profile was evaluated by RT-qPCR using the 2^−ΔCt^ method and the actin gene as an endogenous control. A bar graph showing the expression values in each condition is presented in Fig. S11.

The phenotypic characterization revealed that the Δ*nsd3* strain is more resistant to cell wall stress induced by CR and CFW than the WT (Fig. 2C and D). These results led us to investigate the role of Nsd3 in cell wall remodeling. First, the organization of the cell wall in the WT and Δ*nsd3* strains was analyzed under normal growth conditions (without stress) using transmission electron microscopy (TEM). The results demonstrate that the deletion of *nsd3* affects the organization of the cell wall, causing a significant reduction in cell wall thickness (Fig. 9A and B).

Fungal cell wall is composed of proteins and polysaccharides, including chitin, β-1,3-glucan, and β-1,6-glucan (49). Chitin and β-1,3-glucan are synthesized by chitin synthase and β-1,3-glucan synthase enzymes, respectively. *T. reesei* encodes one β-1,3-glucan synthase (*fks1*) and eight chitin synthases (*chs1-8*) (50, 51). These genes involved in cell wall biosynthesis are not differentially expressed in the RNA-Seq data under any of the conditions analyzed (data not shown). However, our ATAC-Seq data show that the genes *fks1*, *chs3*, and *chs7* present a more accessible promoter in the Δ*nsd3* strain than in the WT (Fig. 9C), indicating that Nsd3 exerts some regulation over them.

To obtain a more comprehensive view of the role of Nsd3 in regulating the maintenance of cell wall integrity, the WT and Δ*nsd3* strains were exposed to CR for 1 h to induce cell wall stress, and the expression profiles of *nsd3*, *tmk2* (the mitogen-activated protein kinase that regulates the cell wall integrity pathway in *T. reesei*), *fks1*, and *chs* genes were analyzed by RT-qPCR (Fig. 9D). First, it was observed that *nsd3* mRNA levels drastically decrease after exposure to CR (*P* < 0.0001), reinforcing that this transcription factor acts as a repressor of cell wall integrity maintenance. Interestingly, the expression of some genes (*tmk2*, *fks1*, *chs4*, *chs3*) decreases in the WT after exposure to stress (*P* < 0.05). In the Δ*nsd3* strain, the expression of these genes is upregulated compared to the WT after exposure to CR (*P* < 0.05). This suggests that Nsd3 represses the expression of *tmk2*, *fks1*, *chs4,* and *chs3* during cell wall stress. An increase in the expression of *chs8* and *chs7* was also observed in the Δ*nsd3* strain after treatment with CR (*P* < 0.01). For the *chs2* and *chs6* genes, a downregulation was observed in the Δ*nsd3* strain after CR exposure (*P* < 0.01). For the *chs5* gene, *nsd3* deletion did not impact its expression during cell wall stress. Taken together, these results indicate that Nsd3 regulates the organization and maintenance of cell wall integrity, affecting chromatin organization in the promoters of genes important for cell wall biosynthesis to repress their expression during cell wall stress.

### Nsd3 regulates the expression of genes involved in secondary metabolism

**Fig 10:**
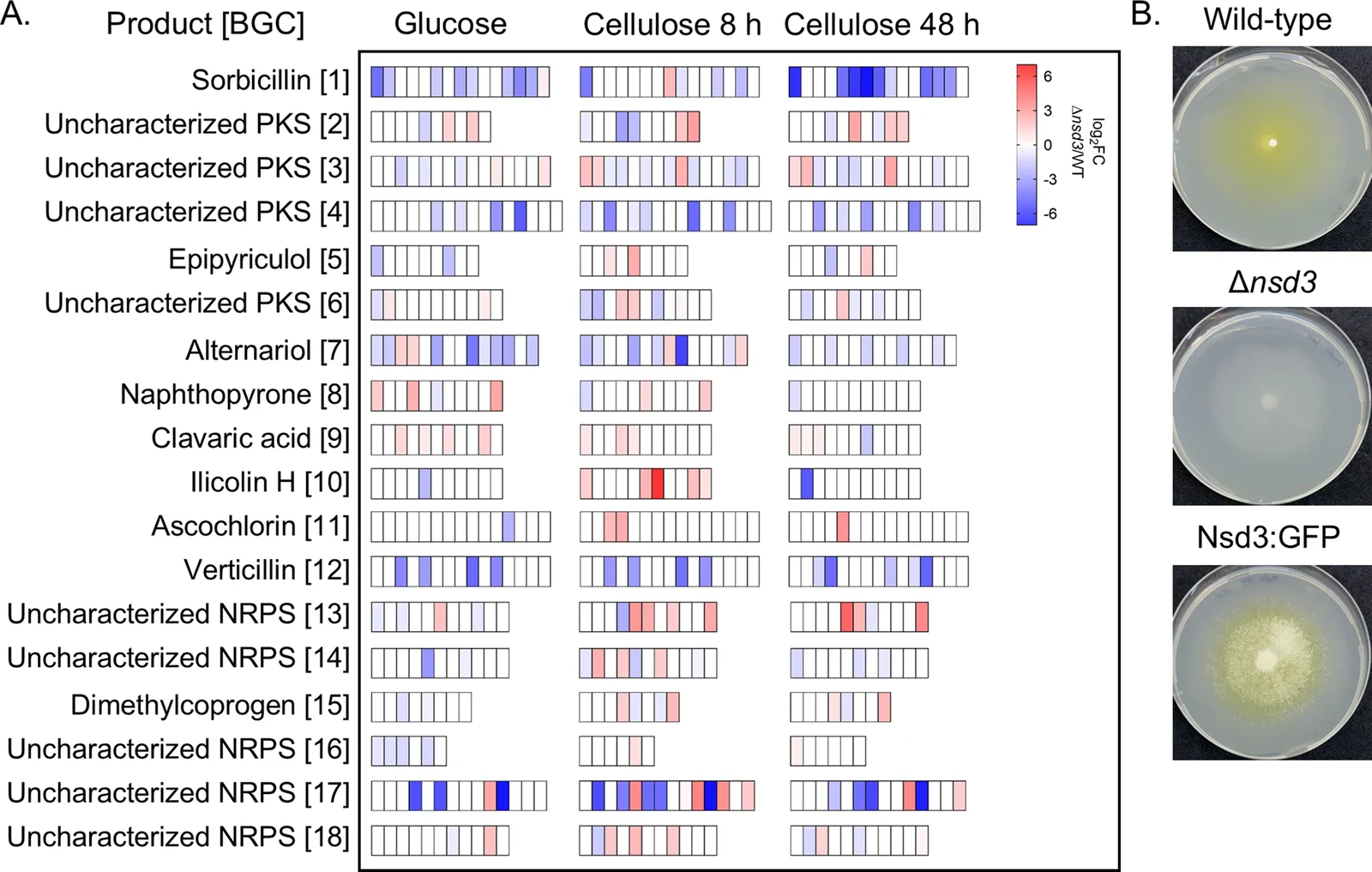
Nsd3 regulates the expression of genes involved in secondary metabolism. (**A**) Heatmap showing gene expression values for each of the 18 BGCs in the analyzed transcriptomes. Values are in log_2_FC. (**B**) Deletion of *nsd3* affects sorbicillinoids production in *T. reesei*. WT, Δ*nsd3*, and Nsd3:GFP strains were cultivated in solid GMM for 5 days, and the pictures were captured. The experiment was performed in triplicate for each strain.

Functional enrichment analysis showed that genes involved in secondary metabolism are enriched among the DEGs in the Δ*nsd3* strain (Fig. 5A, D and E and Fig. S8D). Secondary metabolites are synthesized by large multidomain enzymes called polyketide synthases (PKS), non-ribosomal peptide synthetases (NRPS), and/or NRPS-PKS hybrid systems. These enzyme-encoding genes are located in genomic regions close to genes encoding proteins (transcription factors, transporters, and modifying enzymes) involved in the biosynthesis of their metabolites. These regions are called biosynthetic gene clusters (BGCs) (52). *T. reesei* genome encodes 11 PKS, two NRPS-PKS hybrids, and 10 NRPS (53). Anti-SMASH analysis identified 18 BGCs in its genome, nine of which are PKS type (1–9), two are NRPS-PKS hybrid type (10 and 11), and seven are NRPS type (12–18) (Fig. 10A). The expression of each gene in each of the 18 BGCs was analyzed in the transcriptomes of the Δ*nsd3* strain. The results show that the deletion of *nsd3* impacts the expression of at least one gene in each BGC, with most being downregulated in the mutant (Fig. 9A). BGCs that produce sorbicillin (1), alternariol (7), and verticillin (12) are among the most impacted, along with BGCs 4 and 17, whose products have not yet been characterized (Fig. 10A). The most well-studied cluster in *T. reesei* is the SOR cluster, which produces several yellow pigments called sorbicillinoids. The deletion of *nsd3* causes a reduction in the expression of several genes in this cluster under all conditions analyzed, especially the PKS *sor1* and *sor2* and the regulators *ypr1* and *ypr2*, the main genes of this BGC (54). Plate growth assays showed that the WT and complemented Nsd3:GFP strains produce sorbicillinoids, while the Δ*nsd3* strain fails to do so (Fig. 10B). Taken together, these results indicate that Nsd3 regulates the expression of genes in diverse secondary metabolite clusters and affects the production of sorbicillinoids.

## DISCUSSION

A better understanding of the complex regulatory system behind cellulase production in biomass-degrading fungi is crucial for developing more robust strains to increase enzyme production, improve fermentation efficiency, and reduce the costs of production of value-added products from lignocellulose biomass. In this work, we used an integrative analysis of RNA-Seq data and identified a set of novel transcription factors potentially involved in the regulation of cellulase production in *T. reesei*. Using a similar approach, we previously identified and characterized two transcription factors, Azf1 and Rme1, that directly regulate cellulase expression (55–57).

Our analysis led us to a transcription factor encoded by the gene TRIREDRAFT_75672, a homolog of NsdC, a well-studied transcription factor in various *Aspergillus* species. The NsdC transcription factor was first identified in a screening of *A. nidulans* mutants that failed to undergo sexual development (27). *A. nidulans* is a homothallic fungus, and the deletion of *nsdC* abolishes the formation of cleistothecia, showing that NsdC is essential for sexual reproduction in this fungus (27). This role is conserved in the heterothallic fungus *A. flavus*: deletion of *nsdC* in at least one of the sexual partners impairs sexual reproduction (29). However, in *A. fumigatus*, which is also heterothallic, the absence of *nsdC* in both sexual partners is necessary to abolish sexual reproduction, indicating a slight divergence in the role of NsdC in sexual reproduction in Ascomycetes (30). Our results show that, in *T. reesei*, Nsd3 is not necessary for sexual reproduction, even when it is absent in both sexual partners (Fig. 1C). However, some genes related to sexual development are differentially expressed in the Δ*nsd3* strain, such as the peptide pheromone precursor *hpp1*, the pheromone receptor *hpr2*, and the mating type transcription factor *mat1-2-1*, among others (Fig. S9A). These results show that Nsd3 is not essential for sexual reproduction in *T. reesei*, but it does exert some regulation over genes involved in this process.

Characterization of NsdC in *A. fumigatus* and *A. flavus* showed that it is important for vegetative growth in solid medium. Δ*nsdC* mutants have retarded growth and conidiation defects (29, 30), a phenotype also observed in the Δ*nsd3* strain (Fig. 1A and B), indicating that activation of genes involved in vegetative growth and conidiation is a central role for this transcription factor. In the RNA-Seq data, the gene TRIREDRAFT_112560, encoding a Zn2Cys6 transcription factor, is one of the most downregulated genes in the Δ*nsd3* strain on glucose (log_2_FC = −9.84). This gene has a homolog in *A. nidulans*, SsgI, that is involved in conidiation (42). Our data suggest that Nsd3 and TRIREDRAFT_112560 may interact to regulate conidiation in *T. reesei*. Furthermore, genes potentially involved in asexual development are differentially expressed in the Δ*nsd3* strain, especially the homolog of *flbA*, a transcription factor required for conidiation in *A. nidulans* (58).

Despite being extensively studied for decades, little is known about the stress response in *T. reesei*. Here, we showed that Nsd3 is involved in the response to different stresses (Fig. 2). The Δ*nsd3* strain is more sensitive to osmotic stress caused by sorbitol and NaCl than the WT but is more resistant than the WT to cell wall stress caused by CR and CFW and to oxidative stress induced by menadione. The role of NsdC in the stress response has only been investigated in *A. fumigatus*, where deletion of *nsdC* increases the resistance to stress caused by sorbitol while decreasing tolerance to cell wall stress (30). For the first time in *T. reesei*, the chromatin accessibility profile was assessed by ATAC-Seq. The results showed that *gpd1*, which participates in the response to osmotic stress, presents a more accessible promoter in the Δ*nsd3* strain (Fig. 6E). In addition, other genes involved in glycerol and trehalose metabolism are upregulated in the Δ*nsd3* strain (Fig. S9D). Fungi use these storage molecules to improve intracellular osmolarity in response to osmotic stress (59).

In *A. fumigatus*, NsdC participates in cell wall composition and organization, as well as in calcium tolerance and signaling (30). In *T. reesei*, Nsd3 also participates in the calcium pathway, which had a more positive effect on the mutant strain than on the WT (Fig. 2F and G). Genes encoding calcium transporters (*yvc1*, *yvc3*, *yvc4*, and *vcx1*) are upregulated in the Δ*nsd3* (Fig. S9C), suggesting that the calcium effect on this strain is due to its high uptake by those transporters.

Deletion of *nsd3* affects cell wall organization in *T. reesei*, causing a reduction in cell wall thickness (Fig. 9A and B). In addition, our ATAC-Seq data also show that the promoters of *fks1*, *chs3*, and *chs7* are more accessible in the Δ*nsd3* strain (Fig. 9C), and gene expression analysis showed that, during cell wall stress, genes involved in cell wall biosynthesis are upregulated in the Δ*nsd3* strain (Fig. 9D). These results indicate that Nsd3 recruits chromatin remodelers to the promoters of cell wall biosynthesis genes to repress their expression.

Characterization of Nsd3 in *T. reesei* revealed a new role for this regulator in fungi: it acts as a repressor of cellulase and hemicellulase production. Deletion of *nsd3* resulted in a dramatic increase in the expression of holocellulolytic genes, immediately after induction with cellulose (Fig. 3). This increased expression of genes encoding hydrolases persists for up to 48 h of culture on cellulose. Furthermore, the Δ*nsd3* strain also showed higher levels of specific cellobiohydrolase, β-glucosidase, and β-xylosidase activities than the WT (Fig. 4). Specific CMCase and xylanase activities are also higher in Δ*nsd3*, but significantly decrease after 48 h when compared to the WT (Fig. 4B and D). These results show that Nsd3 represses the expression of *T. reesei* holocellulases, and its deletion causes a fast and robust activation of the expression of holocellulolytic genes, leading to increased synthesis and secretion of these enzymes. This increase, in turn, affects the dynamics of holocellulose hydrolysis and the fungal secretome, reducing the production of endoglucanases and xylanases during biomass degradation. In *A. nidulans*, it is reported that NsdC is important for normal growth in several carbon sources (27); however, its role in cellulase production in other fungi remains to be investigated.

The transcriptional profile of the Δ*nsd3* strain under cellulase production conditions was assessed by RNA-Seq. The results confirm that the regulation exerted by Nsd3 on holocellulase expression is stronger in the early culture times (8 h) and is attenuated after 48 h (Fig. 5F). Functional enrichment analyses show that genes encoding sugar transporters are enriched among the differentially expressed genes in the Δ*nsd3* strain compared to the WT. Sugar transporters play an important role in regulating cellulase production, acting in the uptake of the inducing signal and in the transport of sugars from biomass degradation, such as sophorose and cellobiose, which act as inducers of holocellulase expression (41). The genes encoding the transporters Crt1 and Tr44175, capable of transporting sophorose and cellobiose (9–12), are upregulated in the Δ*nsd3* strain throughout the cellulose culture (Fig. 7B). Crt1 acts as a transceptor and is crucial for the expression of cellulases, where its deletion eliminates cellulolytic production in the fungus (12, 13). The Tr44175 transporter has been characterized by our group, and the results indicate that it is also important for cellulase production (Pereira et al., unpublished data). Our ChIP assay demonstrated stronger Nsd3 occupancy in the *crt1* promoter *in vivo* (Fig. 8D). It is noteworthy that our ChIP results also showed that Nsd3 directly binds to the promoter of the β-glucosidase *cel1b* (Fig. 8G) to activate its expression (log_2_FC = −1.17 in the RNA-Seq). A study by Pang and co-authors showed that overexpression of *cel1b* decreases *crt1* expression and, consequently, decreases the cellobiose uptake by the fungus (60). In addition, ATAC-Seq results show that the *stp1* and *glt1* genes, which are part of the dual-affinity glucose system, are more accessible in the Δ*nsd3* strain (Fig. 7C), and the ChIP assay showed that Nsd3 can bind to the promoter of *stp1 in vivo* (Fig. 8E). The RNA-Seq and ATAC-Seq data, together with the protein-DNA interaction assay, indicate that sugar transporters are important regulatory targets of Nsd3. Further transport assays in the Δ*nsd3* strain are necessary to confirm this hypothesis.

The transcription factor Ace4 is an important activator of cellulase production in *T. reesei*. Ace4 directly binds to the promoters of the cellulolytic genes *cel7a* and *cel6a*, and the TFs *ace3* and *xyr1*, the main activators of cellulase expression (61). The *ace4* gene is upregulated (log_2_FC = 1.45) and exhibits a more accessible promoter in the Δ*nsd3* strain (Data Set S2). Further ChIP assay showed that Nsd3 can directly bind to the promoter of *ace4* (Fig. 8C). These results point to Ace4 as a critical player in the cellulase regulatory network mediated by Nsd3. As Ace4 activates the expression of Ace3 and, concomitantly, they activate the expression of Xyr1, we compared the Δ*nsd3* transcriptome with available transcriptomes from the Δ*ace3* and Δ*xyr1* strains (22, 62). Nsd3 represses the expression of a small set of genes that are targets of these activators, especially Ace3-regulated genes involved in carbohydrate transport and Xyr1-regulated genes involved in carbohydrate metabolism and lipid, fatty acid, and isoprenoid metabolism (Fig. S12).

**Fig 11:**
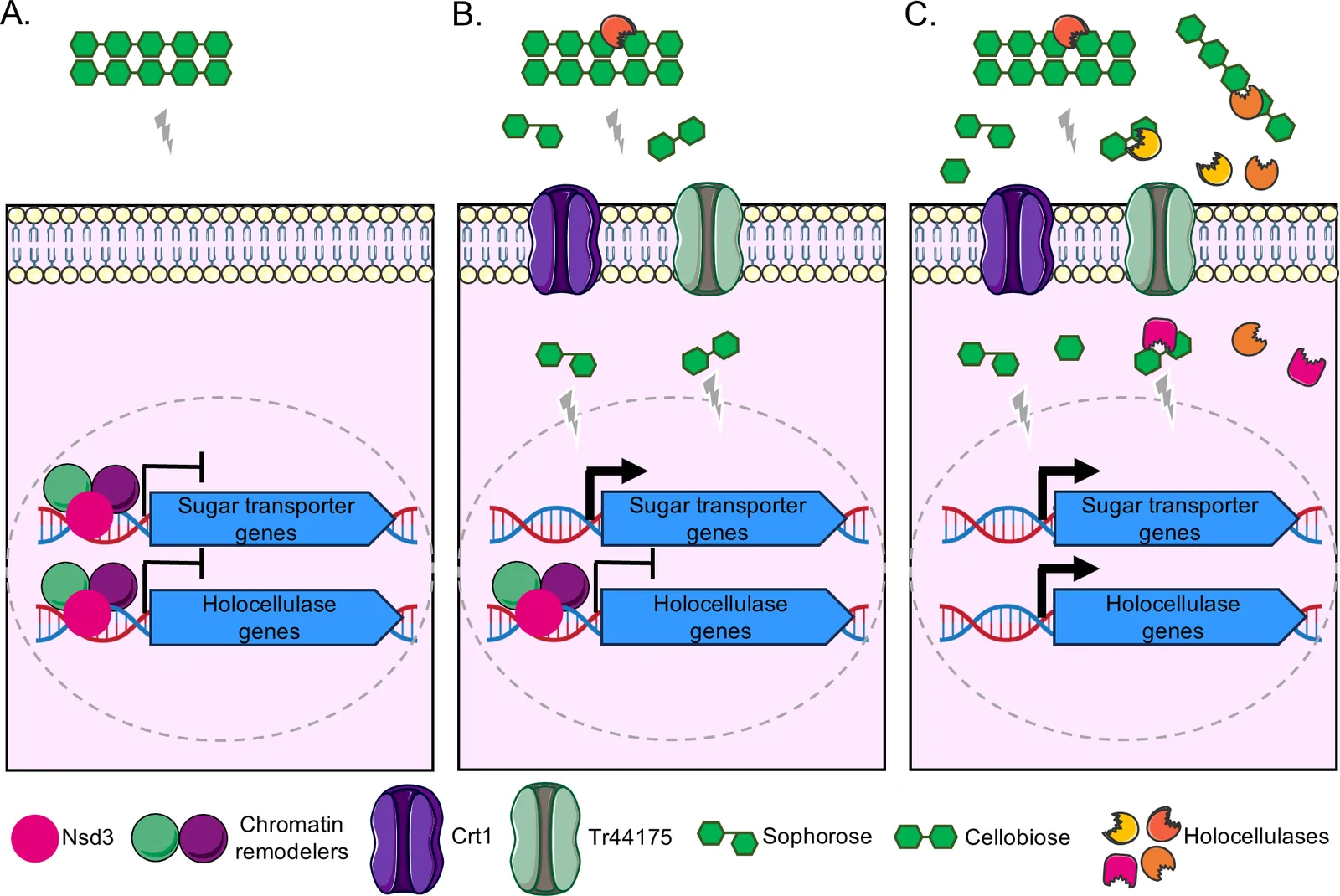
Model for Nsd3-mediated regulation of holocellulase production. (**A**) Nsd3 recruits chromatin remodelers to repress the expression of sugar transporter and holocellulase genes. (**B**) After the fungus senses the plant biomass, Nsd3, and its corepressors leave the promoters of sugar transporter genes, allowing their transcription. These transporters, such as Crt1 and Tr44175, go to the membrane and import sophorose and cellobiose, originating from the basal activity of cellulases, thereby activating holocellulase expression. (**C**) Nsd3 and its corepressors are removed from the promoters of cellulolytic genes, resulting in increased expression and synthesis of holocellulases.

In our proposed model of Nsd3-mediated regulation of holocellulase production, this transcription factor recruits chromatin remodelers to repress the expression of sugar transporter and holocellulase genes (Fig. 11A). After the fungus senses the plant biomass, Nsd3 and its corepressors leave the promoters of sugar transporter genes, allowing their transcription. These transporters, such as Crt1 and Tr44175, go to the membrane and import sophorose and cellobiose, originating from the activity of “scout” cellulases (Fig. 11B). In the intracellular environment, these disaccharides cause the activation of holocellulase expression, and Nsd3 and its corepressors are removed from the promoters of cellulolytic genes, resulting in increased expression and synthesis of holocellulases (Fig. 11C).

Functional enrichment analyses of RNA-Seq data also showed that genes involved in secondary metabolism are enriched among the DEGs in the Δ*nsd3* strain compared to the WT, indicating that the regulation of secondary metabolism is a conserved role with its homolog in *A. flavus*. Inspection of *T. reesei* BGCs revealed that the expression of at least one gene in each cluster is modulated by Nsd3 (Fig. 10A). The effects of *nsd3* deletion on the production of these metabolites remain to be investigated. One of the BGCs most affected by Nsd3 is SOR, which is responsible for the production of yellow pigments, and several genes in this cluster are downregulated, resulting in the absence of sorbicillinoids in the Δ*nsd3* strain (Fig. 10B).

Integrative analysis of RNA-Seq data allowed the identification of a novel regulator of holocellulase production in *T. reesei*. Using functional genomics, biochemical, genetic, genomic, and cell biology approaches, we showed that Nsd3 regulates several biological processes in *T. reesei*. Some functions are conserved in other fungi, such as the regulation of conidiation, stress response, and secondary metabolism. Characterization of Nsd3 also revealed that it represses holocellulase expression. By integrating RNA-Seq and ATAC-Seq data and protein-DNA interaction assay, we propose that this repression is mediated by the regulation of the expression of important sugar transporters, responsible for uptaking the inducer molecules (sophorose and cellobiose) into the cell. The results highlight Nsd3 as a new target for *T. reesei* engineering to design high-performance cellulase production strains.

## MATERIALS AND METHODS

### Identification of DNA motifs and putative regulators of cellulase production in *T. reesei*

For the identification of *cis-*regulatory elements, the analyses were performed as previously described (63), with some adaptations, and the overall workflow is shown in Fig. S1. Nine data sets of differentially expressed genes in RNA-Seq data from *T. reesei* strains QM9414, Δ*xyr1*, and Δ*cre1* grown on cellulose, sophorose, and glucose as sole carbon sources (7, 21, 22, 31) were analyzed. For each data set, a 1.5 kb DNA sequence immediately upstream of ATG was retrieved from the complete genome sequence of *T. reesei,* available at the JGI homepage (http://genome.jgi-psf.org/Trire2/Trire2.home.html), using *ad hoc* Perl scripts. These promoter-region sequences were used for *de novo* motif discovery with MEME (48). Search parameters were set to identify short (6 to 10 bp) unique elements that were overrepresented in the data sets. The sets of identified motifs were compared to the database of *cis*-regulatory elements in *S. cerevisiae* using TOMTOM to identify similar motifs related to previously characterized elements. Next, the protein sequence of each *S. cerevisiae* transcriptional factor associated with these motifs was used to search for homologous genes in *T. reesei* using BLASTp. Finally, the expression of these genes was assessed in RNA-Seq data from the *T. reesei* strain QM6a grown on the lignocellulosic biomass sugarcane bagasse (64) to select the most promising candidates.

### Sequence analysis

Protein sequences of *T. reesei* and other fungi were obtained from JGI, FungiDB, and NCBI. Amino acid sequence alignment was performed using ClustalW, and phylogenetic analysis was performed with MEGA X using the neighbor-joining method with 1,000 bootstraps (65). For structural analysis, protein sequences from *T. reesei*, *A. nidulans*, *A. fumigatus,* and *A. flavus* were submitted to SMART (http://smart.embl-heidelberg.de/).

### Strains and culture conditions

*Escherichia coli* DH5α was used for plasmid propagation. The strain was cultured on LB medium with 100 µg/mL ampicillin at 37°C and 200 rpm.

*T. reesei* TU6 (ATCC MYA-256) and mutant strains were grown in MEX medium (3% [wt/vol] malt extract, 2% [wt/vol] agar) at 30°C for 7–10 days until complete conidiation. For all experiments, 10^6^ conidia/mL of each strain were inoculated in Mandels-Andreotti medium (MAM) (66) containing the respective carbon source, and the cultures were incubated in an orbital shaker (200 rpm) at 30°C for the indicated time. In the experiments using mycelia as inoculum, 10^6^ conidia/mL for each strain were inoculated in MAM containing 1% glycerol (vol/vol), and after 24 h, the mycelium was collected, washed with MAM without a carbon source, and transferred to MAM with 1% cellulose (wt/vol). The experiments were conducted in triplicate for each sample. After induction, the mycelia were collected by filtration, frozen, and stored at −80°C, and the supernatant was collected and stored at −20°C. When necessary, 10 mM uridine was added to the cultures.

### Construction of gRNAs and generation of pCas9-nsd3 and pCas9-pyr4 vectors

For deletion of *nsd3* using the CRISPR-Cas9 system, the pTrCas9gRNA1 plasmid (3), provided by the National Biorenewables Laboratory (LNBR) of the National Center for Research in Energy and Materials (CNPEM), was used as a backbone for cloning the gRNAs. Construction and cloning of the gRNAs were performed as previously described (3), with some adaptations. gRNAs were constructed by fusion PCR of two single-stranded fragments: a 98 nt target-specific fragment (containing the protospacer sequence) and another universal fragment of 158 nt, with a 23 nt overlap between them to allow the fusion. For gRNAs construction, 0.4 µM of each fragment and primers F and R were used for amplification. The DNA was purified and used for a second PCR with primers that contained an overlap sequence to plasmid pTrCas9gRNA1. The product of the second reaction was purified and cloned into plasmid pTrCas9gRNA1, previously linearized with Bsp1407I, using NEBuilder HiFi DNA Assembly Master Mix, generating the plasmid pCas9-nsd3. Construction of the pCas9-pyr4 vector was performed in the same manner, using a 98 nt fragment and specific primers with *pyr4* as a target. All the reactions were performed with Phusion High-Fidelity DNA Polymerase. All the primers used are listed in Table S2. All constructs were confirmed by sequencing.

### Construction of mutant strains

Deletion cassette was constructed by *in vivo* recombination in *S. cerevisiae*. Briefly, 1 kb fragments of the 5′-UTR and 3′-UTR from the *nsd3* locus were amplified from *T. reesei* genomic DNA (gDNA) using the primers Nsd3 5F/R and 3F/R, respectively, along with the selection marker *pyr4*. The purified 5′-UTR, 3′-UTR, and *pyr4* fragments plus the linearized pRS426 vector cut with XhoI and EcoRI were transformed into the *S. cerevisiae* strain SC-9721 (*MATα his3-Δ200 URA3-52 leu2Δ1 lys2Δ202 trp1Δ63*), using the lithium acetate method (67). Deletion cassette was PCR-amplified using the primers 5F and 3R from gDNA extracted from the respective *S. cerevisiae* transformant.

To construct the Nsd3:3×HA:hph and Nsd3:GFP:hph cassettes, a 3 kb fragment encompassing the *nsd3* 5′-UTR and the open reading frame (ORF) without stop codon was amplified from *T. reesei* gDNA using the primers Nsd3 5F and Nsd3 ORF R, along with the 1 kb 3′-UTR DNA fragment, amplified with primers Nsd3 RC 3F and Nsd3 3R. The 3.2 kb linker:3×HA:trpCt:hph and 3.8 kb linker:GFP:trpCt:hph fragments were amplified with the primers pDBM F and pDBM R from the plasmids pDBM1 and pDBM2, respectively. The set of fragments was transformed into the *S. cerevisiae* strain SC-9721 as described above. Cassettes construction was confirmed by PCR using the primers 5F and 3R from gDNA extracted from the respective *S. cerevisiae* transformant. The plasmids containing the cassettes were extracted with Zymoprep Yeast Plasmid Miniprep II, linearized with NheI (New England Biolabs), and used for fungal transformation. All the reactions were performed with Phusion High-Fidelity DNA Polymerase. All the primers used are listed in Table S2.

For the construction of the Δ*nsd3* strain, TU6 protoplasts were obtained by mycelium digestion with lysing enzymes from *Trichoderma harzianum* (Sigma #L1412), and fungal transformation was carried out as previously described (3, 33), with some modifications. For transformation, 3 µg of pCas9-nsd3 vector and 10 µg of linear cassette for genomic integration were used in a total volume of 10 µL. Transformants were plated on MAM containing 2% glucose, 1.2 M sorbitol, and 50 µg/mL hygromycin, without uridine, and incubated at 30°C until colonies were observed. Colonies were then transferred to MAM with 2% glucose, without uridine, and incubated until complete conidiation. For colony purification, they were resuspended in a saline solution and spread on uridine-free MAM plates with 0.1% Triton X-100. After three rounds on Triton plates, transformants were grown in liquid MAM for DNA extraction. *nsd3* deletion was confirmed by diagnostic PCR and RT-qPCR (Fig. S4). Loss of the pCas9-nsd3 vector was confirmed by the absence of growth by the Δ*nsd3* strain in the presence of 50 µg/mL hygromycin.

Nsd3:GFP and Nsd3:3×HA strains were obtained by electroporation due to the growth defects of the Δ*nsd3* strain. For transformation, Δ*nsd3* fresh conidia were harvested in 10 mL of cold 1.2 M sorbitol, filtered with cheesecloth, and washed three times with cold 1.2 M sorbitol at 10,000 rpm for 10 min each. For electroporation, 75 µL of conidia were transferred to a pre-cooled cuvette and mixed with 3 µg of pCas9-pyr4 vector and 10 µg of linear cassette for genomic integration (in a total volume of 7.5 µL), and incubated on ice for 15 min. The system was electroporated at 1.8 kV, 600 Ohms, and 25 µF. Immediately after electroporation, 330 µL of cold 1.2 M sorbitol was added to the cuvette. The suspension was transferred to conical tubes containing 170 µL of YPD (1% yeast extract, 2% peptone, and 1% glucose) and incubated overnight at room temperature. Transformants were plated on MEX containing 100 µg/mL hygromycin and incubated at 30°C until colonies were observed. Colony purification was performed as described before, except that MEX with hygromycin was used as the selective medium. Positive transformants were confirmed by diagnostic PCR, and detection of fusion proteins was performed by Western blot (Fig. S5). Loss of the pCas9-pyr4 vector was confirmed by diagnostic PCR.

### Conidium count

Fresh conidia of the WT and Δ*nsd3* strains were inoculated onto solid MEX at 30°C for 8 days. After this period of growth, all conidia on the plates were harvested and resuspended in 1 mL of a solution containing 0.8% NaCl and 0.05% Tween 80 and shaken for 10 min at 2,000 rpm. Subsequently, the conidia were filtered through glass wool filters at 3,000 rpm for 4 min and counted using a Neubauer chamber. The assay was performed in triplicate. Statistical tests were performed using a *t*-test with Welch’s correction.

### Sexual crosses and screening of the offspring

All crosses were performed on 3% MEX agar plates at 22°C and 12 h light–dark cycles as previously described (24). After this period, ascospores were collected from the plate lid using sterile H_2_O and spread onto MEX plates with 0.1% Triton X-100 and incubated at 30°C until colonies appeared. Single colonies were transferred to new MEX plates and grown at 30°C. The gDNA from each offspring was extracted and used in PCRs to verify the mating type and the presence of the *nsd3* gene. Primers are listed in Table S2.

### Sensitivity test to different stressors

To test the stress response, 10 µL of a solution containing 10^6^ conidia/mL from the wild-type or mutant strains were inoculated on the center of MM plates [1 g/L MgSO_4_.7H_2_O, 10 g/L KH_2_PO_4_, 6 g/L (NH_4_)_2_SO_4_, 3 g/L Na_3_C_6_H_5_O_7_, 20 mL/L trace elements, 20 g/L agar] with 2% glucose in the absence or presence of different concentrations of CFW (20 and 40 µg/mL), CR (50 and 100 µg/mL), NaCl (0.2, 0.5, and 1 M), menadione (20 and 40 µM), sorbitol (0.2, 0.5 and 1 M), CaCl_2_ (10, 50, and 100 mM), and EGTA (5 and 10 mM). After incubation for 5 days at 30°C, the mycelial diameter under the indicated conditions was recorded. Three replicates of each experiment were performed. Statistical tests were performed using a *t*-test with Welch’s correction to compare the growth of the strains.

### Western blot

*T. reesei* strains were grown in MAM with glucose for 24 h. The mycelia were collected and added to 500 µL of protein extraction buffer (50 mM HEPES pH 7.5, 150 mM NaCl, 1 mM EDTA, 0.02% NP-40, 1 mM PMSF, one tablet of Roche complete mini protease inhibitor cocktail). The samples were sonicated (30% amplitude, 1 s on, 1 s off) and then centrifuged at 13,000 rpm for 5 min at 4°C. The supernatant was collected into a new tube. Protein concentration was determined using the Qubit Protein Assay Kit (Thermo Fisher Scientific). The same amount of protein from each sample (20 µg) was subjected to SDS-PAGE and Western blot. Membranes were probed with rat anti-HA (Roche #11867431001, 1:5,000 dilution) or mouse anti-GFP (Santa Cruz Biotechnology #sc9996, 1:10,000 dilution) primary antibodies, and with the horseradish peroxidase (HRP)-conjugated anti-rat (Thermo Fisher Scientific #31470, 1:20,000 dilution) or anti-mouse (Thermo Fisher Scientific #32430, 1:10,000 dilution) secondary antibodies. A Western blot for actin (68) was performed as a control.

### Gene expression analysis by RT-qPCR

*T. reesei* mycelia were macerated, and the RNA was extracted using TRI (Sigma-Aldrich), according to the manufacturer’s instructions. For cDNA synthesis, 1 µg of RNA was first treated with DNAse I (Thermo Fisher Scientific) to remove genomic DNA. After this step, cDNAs were synthesized using Maxima First Strand cDNA Synthesis (Thermo Fisher Scientific), according to the manufacturer’s instructions. They were diluted 50× and used for RT-qPCR analysis in Bio-Rad CFX96 equipment together with SsoFast EvaGreen Supermix (Bio-Rad), according to the manufacturer’s instructions. Gene expression levels were calculated from the threshold cycle according to the 2^−ΔCT^ method, relative to transcript levels of β-Actin (69, 70). The amplification program used in this study was as follows: 95°C for 10 min followed by 39 cycles of 95°C for 10 s and 60°C for 30 s followed by a dissociation curve of 60°C to 95°C with an increment of 0.5°C for 10 s per increment. The primers used for amplification of identified genes are described in Table S3. Statistical tests were performed using a *t*-test with Holm-Sidak’s correction to compare the gene expression levels of the strains.

### Protein and enzyme assays

Protein concentration in the supernatant was determined by the Bradford method, using Bovine Serum Albumin (BSA) as a standard. Carboxymethylcellulase (CMCase) activity was determined by adding 30 µL of 1% carboxymethyl cellulose (CMC) prepared in sodium acetate buffer (pH 4.8) and 30 µL of the sample. The reaction mixture was incubated at 50°C for 30 min. Then, 60 µL of dinitrosalicyclic acid (DNS) was added to the reaction mixture, and the mixture was heated at 95°C for 5 min. For analysis of xylanase activity, 25 µL of the sample was incubated with 50 µL of 1% xylan and incubated at 50°C for 10 min. Then, 75 µL of DNS was added to the reaction mixture and heated at 95°C for 5 min. For both CMCase and xylanase activities, samples were read at an absorbance value of 540 nm. β-glucosidase activity was determined by the addition of 50 µL of 50 mM sodium acetate buffer (pH 5.5), 10 µL of the sample, and 40 µL of 5 mM *p*-nitrophenyl (PNP)-glucoside substrate. The reaction mixture was incubated at 50°C for 15 min, followed by the addition of 100 µL of 1 M sodium carbonate. For analysis of β-xylosidase activity, 50 µL of 50 mM sodium acetate (pH 4.8) was incubated with 10 µL of the sample and 40 µL of 5 mM PNP-xyloside. The reaction mixture was incubated at 50°C for 15 min, followed by the addition of 100 µL of 1 M sodium carbonate. For analysis of cellobiohydrolase activity, 50 µL of 50 mM sodium citrate (pH 4.8) was incubated with 10 µL of the sample and 40 µL of 5 mM PNP-cellobioside. The reaction mixture was incubated at 50°C for 3 h, followed by the addition of 100 µL of 1 M sodium carbonate. For analysis of β-glucosidase, β-xylosidase, and cellobiohydrolase activity, samples were read at an absorbance of 405 nm. One enzyme unit was defined as the amount of enzyme capable of releasing 1 µmol of reducing sugar or hydrolyzing 1 µmol of substrate per min. Statistical tests were performed using one-way analysis of variance (ANOVA) followed by Tukey’s test to compare the enzymatic activity levels of the strains.

### Sensitivity to 2-DG

To test the sensitivity to 2-DG, 10 µL of a solution containing 10^6^ conidia/mL from the WT, Δ*nsd3,* or Δ*cre1* (17, 21) strains were inoculated at the center of MM plates containing 1% cellobiose and increasing concentrations of 2-DG (0.02–0.08%). The plates were incubated for 3 days at 30°C, and then, the pictures were registered. The assay was performed in triplicate.

### RNA-Seq

WT and Δ*nsd3* strains were cultivated on MAM containing glucose or glycerol for 24 h, or cellulose for 8 and 48 h (after pregrowing on glycerol for 24 h). Mycelia were harvested, flash-frozen, and ground with liquid N_2_, and the RNA was extracted using TRI (Sigma-Aldrich), according to the manufacturer’s instructions. RNA was treated with DNAse I (Thermo Fisher Scientific) and purified with the RNeasy Kit (Qiagen), according to the manufacturer’s instructions. RNA integrity was analyzed using an Agilent 2100 Bioanalyser system, and only samples with an RNA Integrity Number (RIN) ≥ 7 were submitted to library preparation. Library preparation was carried out using the Illumina TruSeq Stranded mRNA Library Preparation Kit, according to the manufacturer’s instructions. Samples were sequenced on an Illumina NextSeq 2000 instrument. This experiment was performed in triplicate for each sample and condition.

TrimGalore v0.6.7 (https://github.com/FelixKrueger/TrimGalore) (--paired --quality 20 --phred33 --length 50) was used to filter and trim raw reads, removing Illumina adapters, sequencing artifacts, and reads shorter than 50 bases. The resulting reads were mapped to the *T. reesei* reference genome (http://genome.jgi-psf.org/Trire2/Trire2.home.html) using HISAT2 v2.2.1 (71). featureCounts (72) and the gff3 annotation file from the reference genome were used to calculate raw gene counts. Specifically, we only used primary final gene counts (-k 1). To calculate the differential expression, we fit a generalized (negative binomial) linear model using the R package DESeq2 (73), using the raw number counts per gene as input for each sample. To account for errors in modeling, we calculated a Benjamini–Hochberg (false discovery rate, FDR) adjusted *P*-value for each transcript using Wald tests and used a threshold of FDR-adjusted *P*-value < 0.05. We normalized each WT sample with its respective mutant sample using the contrast function from DESEq2. Principal component analysis (PCA) and hierarchical clustering were performed in FungiExpresZ (74). Functional enrichment analyses were performed in FungiFun3 (75), using functional categorization (FunCat) terms to classify differentially expressed genes (https://fungifun3.hki-jena.de/).

### ATAC-Seq

ATAC-Seq was performed as previously described (76), with some adaptations. *T. reesei* WT and Δ*nsd3* strains were grown on MAM containing glucose for 24 h. After this period, the mycelia were collected, washed with PBS, and transferred to small Petri dishes containing 2 mL of nuclear lysis buffer (10 mM Tris-HCl pH 7.5, 10 mM NaCl, 3 mM MgCl_2_, 0.1% NP-40, 0.1% Triton-X 100, 0.01% digitonin). Nuclei were isolated by chopping and passing the lysate through Miracloth. Samples were centrifuged at 1,000 × *g* for 1 min to pellet cell debris. The supernatant was transferred to another tube and centrifuged at 10,000 × *g* for 1 min. The supernatant was discarded, and the nuclei were washed with 1 mL of wash buffer (10 mM Tris-HCl pH 7.5, 10 mM NaCl, 3 mM MgCl_2_, and 0.01% Tween 20). Nuclei were centrifuged at 10,000 × *g* for 1 min, and the supernatant was discarded. The pellet containing the nuclei was resuspended in 50 µL of transposition buffer (5 mM TAPS-NaOH, 2.5 mM MgCl_2_, 0.33× PBS, 0.1% Tween 20, 0.01% digitonin) containing the Tn5 transposase pre-loaded with Illumina sequencing adaptors (77). The reaction was incubated at 37°C for 30 min, and DNA was purified with the Qiagen MinElute Kit. Libraries were prepared as previously described in (78) and sequenced on an Illumina NovaSeq X Plus Instrument. This experiment was performed in biological replicates for each strain.

TrimGalore v0.6.7 (https://github.com/FelixKrueger/TrimGalore) was used to filter the reads, removing low-quality reads and sequences related to adapters. The resulting reads were mapped to the reference genome (http://genome.jgi-psf.org/Trire2/Trire2.home.html) using BWA v0.7.17 mem (option -M) (79). Reads were sorted and indexed using SAMtools v1.16.1 (80). BigWig files were created using deepTools v3.5.2 (81), module “bamCoverage,” and normalized by BPM (bins per million mapped reads). The resulting BigWig files were visualized on the Integrative Genomics Viewer (IGV) platform and used to create figures. Peak calling was performed using MACS3 (82), with a *q*-value < 0.01. HOMER (83) was used for genomic annotation and motif analysis. Peaks were filtered to include only those located in proximal promoter regions (between −2,000 and + 500 bp relative to the transcription start site [TSS] of the corresponding gene) to identify genes with accessible chromatin. The final list of accessible genes in the WT and Δ*nsd3* strains is provided in Data Set S2. Functional enrichment analyses were performed in FungiFun3 (75), using functional categorization (FunCat) terms to classify the accessible genes (https://fungifun3.hki-jena.de/).

### Prediction of Nsd3-binding motif and search for its putative targets

The putative Nsd3-binding motif was obtained from the online server at http://zf.princeton.edu/index.php (47). Promoter sequences comprising 2 kb upstream of the ATG were obtained using an *ad hoc* script. Searches for putative Nsd3-binding sites in the promoters of the target genes were performed using FIMO (84), available on MEME (48), to identify possible Nsd3 direct targets.

### Chromatin immunoprecipitation coupled with qPCR (ChIP-qPCR)

The Nsd3:3×HA and WT strains were grown in MAM supplemented with 1% cellulose for 6 h after previous growth on glycerol for 24 h. Mycelia were collected with Miracloth, washed with PBS, and transferred to new flasks containing 10 mL of PBS. Cross-linking was performed with 1% (vol/vol) formaldehyde for 20 min at room temperature before quenching the formaldehyde with 125 mM glycine at room temperature. Mycelia were collected with Miracloth, washed with PBS, and frozen with liquid nitrogen. Mycelia were ground to a fine powder and resuspended in 1 mL of ChIP lysis buffer (CLB: 50 mM HEPES pH 7.5, 140 mM NaCl, 1 mM EDTA, 1% Triton X-100, 0.1% deoxycholate, 1 mM PMSF, one tablet of Roche complete protease inhibitor cocktail). Samples were sonicated for three cycles of 10 min (30 s on and 30 s off, 50% amplitude) before being centrifuged at 14,000 rpm for 5 min at 4°C. Supernatants were collected, and 30 µL were saved as input. Immunoprecipitations (IP) were carried out by first ligating 5 µg of anti-HA monoclonal antibody (Sigma-Aldrich, #H3663), diluted in 200 µL of PBS–0.01% Tween 20, to 20 µL of magnetic Dynabeads Protein A (Invitrogen) for 10 min at room temperature. Subsequently, beads were washed once with 200 µL of PBS-Tween before being resuspended in 50 µL CLB. The remaining supernatants were mixed with the beads for each sample. Samples were incubated at 4°C with rotation for 2 h before the beads were washed as described previously (78). Sample elution and reverse cross-linking were carried out as described previously (78). Samples were treated with RNAse A and Proteinase K, and the DNA was purified with Illustra GFX PCR DNA Purification Kit (GE Healthcare) and eluted in 30 μL of H_2_O. Subsequently, 1 µL of DNA was used for qPCR in Bio-Rad CFX96 equipment using SsoFast EvaGreen Supermix (Bio-Rad), according to the manufacturer’s instructions. The amplification program used in this study was as follows: 95°C for 10 min, followed by 39 cycles of 95°C for 10 s and 60°C for 30 s, followed by a dissociation curve from 60°C to 95°C, with an increment of 0.5°C every 10 s. Primers used in the qPCR are described in Table S4. DNA enrichment at each gene promoter site was calculated using the percent input method, and positive controls included cross-linked but not immunoprecipitated samples (input). All experiments were carried out in biological triplicate. Statistical tests were performed using a *t*-test with Welch’s correction.

### Transmission electron microscopy (TEM)

The WT and Δ*nsd3* strains were grown in MAM with 2% (wt/vol) glucose statically for 18 h at 30°C. Mycelia were collected by centrifugation at 5,000 rpm for 5 min, washed with 1× phosphate-buffered saline (PBS), and immediately fixed in 0.1 M sodium phosphate buffer (pH 7.4) containing 2% (vol/vol) glutaraldehyde and 4% (wt/vol) paraformaldehyde for 24 h at 4°C. Samples were subjected to fixation (1% OsO4), contrasting (1% uranyl acetate), ethanol dehydration, and a two-step infiltration process with Spurr resin (Electron Microscopy Sciences) of 24 at room temperature (RT). Additional infiltration was provided under vacuum at RT before embedment in BEEM capsules (Electron Microscopy Sciences) and polymerization at 60°C for 24 h. Semithin (0.5 µm) survey sections were stained with toluidine blue to identify the areas of best cell density. Ultrathin sections (60 nm) were prepared and stained again with uranyl acetate (1%) and lead citrate (2%). TEM images were obtained using a Jeol JEM-1400 Flash electron microscope at an acceleration voltage of 120 kV using TEM Center Ver1.7.30.3157 software. Cell wall thicknesses of ~ 50 sections of different germlings were measured at ×23,500 magnification, and images were analyzed with the ImageJ software. Statistical tests were performed using a *t*-test with Welch’s correction.

## Data Availability

RNA-Seq and ATAC-Seq data used in this study were deposited in the Gene Expression Omnibus (GEO) at the National Center for Biotechnology Information (NCBI) and are accessible through GEO series accession numbers GSE328452 and GSE327621, respectively. Strains and plasmids are available upon request.

## SUPPLEMENTAL MATERIAL

Supplemental material aem.00865-26-s0001.docx Fig. S1 to S12; Tables S1 to S4.

Data Set S1 aem.00865-26-s0002.xlsx RNA-Seq data.

Data Set S2 aem.00865-26-s0003.xlsx ATAC-Seq data.

ASM does not own the copyrights to Supplemental Material that may be linked to, or accessed through, an article. The authors have granted ASM a non-exclusive, world-wide license to publish the Supplemental Material files. Please contact the corresponding author directly for reuse.
