# Supplementary material for: Trichoderma reesei Nsd3 transcription factor: pleiotropic roles in development, stress response, secondary metabolism, and cellulase production
Source: Appl Environ Microbiol. 2026 Sep 3;92(9):e00865-26. doi: 10.1128/aem.00865-26 (PMC13599534; doi:10.1128/aem.00865-26)
Supplement: Supplementary file 1 [file aem.00865-26-s0001.docx]

**Supplemental Material for**

***Trichoderma reesei* Nsd3 transcription factor: pleiotropic roles in development, stress response, secondary metabolism, and cellulase production**

David Batista Maués^1^, Lucas Matheus Soares Pereira^1^, Andrei Stecca Steindorff^2^, Amanda Cristina Campos Antoniêto^1^, Rafael Silva-Rocha^3^, Marcelo Damário Gomes^1^, Zachary A Lewis^4^, Roberto N Silva^1,5*^

^1^Department of Biochemistry and Immunology, Ribeirão Preto Medical School, University of São Paulo, Ribeirão Preto, São Paulo, Brazil

^2^US Department of Energy Joint Genome Institute, Lawrence Berkeley National Laboratory, Berkeley, California, USA

^3^ByMyCell Inova Simples LTDA, Ribeirão Preto, São Paulo, Brazil

^4^Department of Microbiology, University of Georgia, Athens, Georgia, USA

^5^National Institute of Science and Technology in Human Pathogenic Fungi, São Paulo, Brazil

*Address correspondence to Roberto N Silva, rsilva@fmrp.usp.br

**Fig. S1.** Workflow representing the approach used in this study to identify novel TFs regulating cellulase production in *T. reesei*. RNA-Seq data from *T. reesei* strains QM9414, Δ*xyr1* and Δ*cre1* grown in different carbon sources [cellulose (Cell), sophorose (Soph) and glucose (Glu)] were assessed to identify putative *cis*-regulatory elements enriched in the promoter of genes differentially expressed in these conditions using MEME. The sets of identified motifs were compared to the database of *cis*-regulatory elements in *Saccharomyces cerevisiae* using TOMTOM. Next, the protein sequence of each *S. cerevisiae* transcriptional factor associated with these motifs was used to search for homologous genes in *T. reesei* using BLASTp. This figure is based on (1).

**Fig. S2.** Enriched motifs and the *S. cerevisiae* transcription factors associated with them. Five motifs (left panel) were found enriched in the promoters of genes differentially expressed in RNA-Seq data. Using TOMTOM, these motifs were associated with characterized *cis*-regulatory elements from *S. cerevisiae* transcription factors (middle panel). These five TFs are involved in several biological processes in *S. cerevisiae* (right panel)*.*

**Fig. S3.** *TRIREDRAFT_75672* encodes the NsdC homolog of Aspergilli. (A) Phylogenetic analysis of *TRIREDRAFT_75672* (highlighted in red) and its orthologs in other fungi. Amino acid sequence alignment was performed using ClustalW, and the cladogram was constructed using the MEGA X tool, employing the Maximum-Likelihood method and the Poisson model, with 1,000 bootstraps as a parameter. (B) Structural analyses of Nsd3 and its orthologs in *A. nidulans, A. fumigatus,* and *A. flavus.* The DNA-binding domain is composed of three zinc fingers. Protein sequences were subjected to SMART for domain analysis. (C) Alignment between the sequences of Nsd3 and NsdC, its homolog in *A. nidulans*, in the DNA-binding domain (black and gray boxes). Cysteine and histidine residues are highlighted in red. The black box indicates the C_2_H_2_ zinc finger, and the gray box indicates the C_2_HC-type zinc finger.

**Fig. S4.** Construction of the Δ*nsd3* strain. (A) Schematic representation of the *nsd3* deletion in the *T. reesei* genome and the set of PCRs to confirm the deletion. The CRISPR-Cas9 system was used to delete the gene, and the protospacer sequence is indicated in the diagram. Transformants and the parental strain (TU6) were subjected to three conventional PCRs to confirm the gene deletion and correct cassette integration (B-D). To verify the presence of the *nsd3* ORF, reaction R1 was performed, and 6 transformants were positive for the deletion (B). Correct cassette integration in the 5' (C) and 3' (D) regions in the gene locus was confirmed by reactions R2 and R3, respectively. A DNA-free (H_2_O) reaction was performed as an additional negative control. (E) RT-qPCR showing *nsd3* expression in WT and 4 transformants after growth in MAMwith glucose for 24 h. *nsd3* expression was not detected in any of the transformants analyzed.

**Fig. S5.** Construction of Nsd3:GFP and Nsd3:3xHA strains. (A) Amplification of the Nsd3:GFP and Nsd3:3xHA cassettes, constructed by homologous recombination in yeast. (B) Schematic representation of the complementation cassettes and the set of PCRs to verify the insertion into the fungal genome. The complementants and the WT and knockout strains were subjected to two conventional PCRs to confirm cassette integration and verify correct insertion into the target gene locus. (C) PCR showing that the cassettes were integrated into the fungal genome. Plasmids containing the constructs were used as a positive control of the reaction (Ctrl), while the DNAs of the WT (TU6) and knockout strains were used as a negative control. A DNA-free reaction (H_2_O) was performed as an additional negative control of the reactions. (D) PCR showing the integration of the cassette into the target gene locus. The DNAs of the WT (TU6) and knockout strains were used as a negative control, and a DNA-free reaction (H_2_O) was performed as an additional negative control for the reactions. (E-F) Western blot for detection of Nsd3:GFP (E) and Nsd3:3xHA (F) proteins. Strains were grown in MAM with glucose for 24 h for protein extraction. A Western blot for actin was performed as a positive control.

**Fig. S6.** Recovery and characterization of the DMx1-11 strain. DMx1-11 (Δ*nsd3, mat1-1*) was obtained from crosses between the Δ*nsd3* (*mat1-2*) and CBS1-1 (*mat1-1*) strains. (A-B) Genotyping the DMx1-11 strain to verify the absence of *nsd3* ORF (A) and the presence of the *mat1-1* locus (B). DNAs from the strains TU6, Δ*nsd3*, and CBS1-1 were used as controls, along with a DNA-free reaction (H_2_O) as a negative control. (C) Phenotype of the DMx1-11 strain. Strains were cultivated on MEX medium for 5 days at 30°C, and then the pictures were taken. (D) Cellulase activities in the DMx1-11 strain. Cellulolytic and hemicellulolytic activities of WT, Δ*nsd3*, and DMx1-11 strains after growth on cellulose for 48 h (after pregrowing on glycerol for 24 h). Culture supernatants were collected at the indicated time and used to quantify cellobiohydrolase, CMCase, β-glucosidase, xylanase, and β-xylosidase. Results are the means of three biological replicates for each strain and error bars indicate standard deviation. Asterisks indicate significant differences (*p ≤ 0.05, **p ≤ 0.01, ***p ≤ 0.001, ****p ≤ 0.0001, One-way ANOVA with a Tukey post-hoc test).

**Fig. S7.** Nsd3 is not involved in CCR. WT, Δ*nsd3,* and Δ*cre1* strains were grown in the presence of cellobiose and increasing concentrations of 2-DG (0.02-0.08%). Pictures were registered after 3 days of culture.

**Fig. S8.** RNA-Seq for the Δ*nsd3* strain. (A) Schematic representation of the experimental workflow for RNA-Seq of the Δ*nsd3* strain. The WT and Δ*nsd3* strains were grown on glucose and glycerol for 24 h and on cellulose for 8 and 48 h (after prior growth on glycerol for 24 h) for RNA extraction. The experiments were performed in triplicate for each sample. (B) Principal-component analysis (PCA) of RNA-Seq samples. (C) Hierarchical clustering heat map to obtain an overview of the differences and similarities of gene expression from each sample based on sample distance. (D) Functional enrichment analysis using FunCat terms for differentially expressed genes in the Δ*nsd3* strain relative to the WT strain on glycerol. An adjusted p-value < 0.05 was used to filter the terms.

**Fig. S9.** Transcription profile of genes involved in sexual development (A), asexual development (B), calcium signaling (C), and stress response (D) in the transcriptomics of the Δ*nsd3* strain. Values are in log_2_FC.

**Fig. S10.** ATAC-Seq analysis in the Δ*nsd3* strain. (A) Genome Browser screenshots showing the ATAC-Seq enrichment signal for WT (blue) and Δ*nsd3* (purple) strains in the promoter of the hexokinase (*hxk*) and cellobiohydrolase *cel7a* genes. *hxk* and *cel7a* promoters served as the positive and negative controls, respectively. (B) The output of *de novo* motif analysis by HOMER on accessible regions in the Δ*nsd3* strain. Motif 1 is shown in Fig 6C.

**Fig. S11.** Nsd3 affects the expression of genes involved in cell wall integrity. The WT and Δ*nsd3* strains were grown in MAM with glucose for 24 h, and then the cells were treated with CR 100 µg/mL for 1 h. Untreated cells served as the control. (A-J) Expression profile of genes involved in cell wall biosynthesis, assessed by RT-qPCR, using the 2^-ΔCt^ method. The β-actin gene was used as an endogenous control. Asterisks indicate significant differences (**p* ≤ 0.05, ***p* ≤ 0.01, ****p* ≤ 0.001, *****p* ≤ 0.0001), as assessed by One-way ANOVA followed by Tukey’s test.

**Fig. S12.** Nsd3 represses Ace3- and Xyr1-regulated genes. (A-B) Venn diagram comparing upregulated genes in the Δ*nsd3* strain with downregulated genes in Δ*ace3* (A) and Δ*xyr1* (B) strains. Shared genes were submitted to functional enrichment analyses using Funcat terms. The significant biological processes are highlighted in the boxes (adjusted p-value < 0.05). RNA-Seq data from Δ*ace3* and Δ*xyr1* strains were retrieved from (2, 3).

**Table S1.**

Gene expression analysis of putative MOT3 homologs in RNA-Seq data from the *T. reesei* QM6a strain after induction with sugarcane bagasse (SCB).(4)

| **Mot3p putative homolog ID** | **Log_2_FC**  **(SCB/Glycerol)** | **Adjusted *p*-value** |
| --- | --- | --- |
| 58897 | 0,29853487 | 0,07697353 |
| 75672 | 1,390766566 | 0,001832774 |
| 112342 | 0,093225493 | 0,696862846 |
| 36543 | 0,038871426 | 0,875290618 |

**Table S2.**

Primers used in this study for conventional PCRs

| **Primer** | **Sequence (5’-3’)** | **Goal** |
| --- | --- | --- |
| Nsd3 5F | GTAACGCCAGGGTTTTCCCAGTCACGACGGCTAGCTGGCCGTTATTAGG | Construction of deletion cassette |
| Nsd3 5R | AACCCAGACAAGACAAGGCAAGTGCAGTCGCAAAGGTGGGAA |  |
| Nsd3 3F | GAAACGGACGGCAACAATCAACCGGGACCCTCCTACTGGTTTCT |  |
| Nsd3 3R | GCGGATAACAATTTCACACAGGAAACAGCCCATGATGGTTGGGTTCGCT |  |
| Nsd3-Pyr4 F | TTCCCACCTTTGCGACTGCACTTGCCTTGTCTTGTCTGGGTT |  |
| Nsd3-Pyr4 R | AGAAACCAGTAGGAGGGTCCCGGTTGATTGTTGCCGTCCGTTTC |  |
| Nsd3 gRNA 98* | AGATTCTGTACAAAGGGCCTGATGAGTCCGTGAGGACGAAACGAGTAAGC TCGTC**GCCCTTGACGTCGACCTCCA**GTTTTAGAGCTAGAAATAGCAAG | Construction of gRNA for *nsd3* |
| Nsd3 gRNA F | AGATTCTGTACAAAGGGCCTGA |  |
| Pyr4 gRNA 98* | AGATTCTGTACAGCGCGCCTGATGAGTCCGTGAGGACGAAACGAGTAAGC  TCGTC**GCGCGCCAGCGACGCCAGCT**GTTTTAGAGCTAGAAATAGCAAG | Construction of gRNA for *pyr4* |
| Pyr4 gRNA F | AGATTCTGTACAGCGCGCCTGA |  |
| gRNA 158 | TCAATGTACAGTCCCATTCGCCATGCCGAAGCATGTTGCCCAGCCGGCGCCAGCGA GGAGGCTGGGACCATGCCGGCCAAAAGCACCGACTCGGTGCCACTTTTTCAAG TTGATAACGGACTAGCCTTATTTTAACTTGCTATTTCTAGCTCTAAAAC | Construction of gRNAs |
| gRNA R | TCAATGTACAGTCCCATTCGCCATGCCG |  |
| Nsd3 gRNA Gibson F | AGCCACCTGAAATAGCTTCTGTACAAAGGGCCTGATGAGTCCGTGAGGA | Cloning *nsd3* gRNA |
| Pyr4 gRNA Gibson F | AGCCACCTGAAATAGCTTCTGTACAGCGCGCCTGATGAGTCCGTGAGGA | Cloning *pyr4* gRNA |
| gRNA Gibson R | CCTCATCCACTCCAAGTCAATGTACAGTCCCATTCGCCATGCCGAAGCATG | Cloning gRNAs |
| Nsd3 ORF R | ACCACCGCTACCACCTCCTCCCGCAGGCACCCCTTCA | Construction of recomplementation cassettes |
| pDBM F | GGAGGTGGTAGCGGTGGT |  |
| pDBM R | GGAATACCCCAGTCCAGATCATG |  |
| Nsd3 RC 3F | CGCCGGCCGAAACTTTTTTACAGGACCCTCCTACTGGTTTCT |  |
| Screening ORF Nsd3 F | AGTTGTAGCACCAGCACCA | Screening of Δ*nsd3* transformants |
| Screening ORF Nsd3 R | TTGTCGTATGCGATGCCC |  |
| Dir Nsd3 F | GCCGTGCTTCGTATGTACT |  |
| Dir Pyr4 R | GGTTGAGCCGTTCAGGTACAGT |  |
| Dir Pyr4 F | TCGTGTCCAAGCGAACAGAG |  |
| Dir Nsd3 R | GTCATGCAGTGGAAGAAGACG |  |
| Screening trpCt R | GCTGACATCGACACCAACGATC | Screening of Nsd3:GFP and Nsd3:3xHA transformants |
| Genotyping mat1-1 F | TCCTCTCAATGCGTTCATGGC | Genotyping *mat1-1* locus |
| Genotyping mat1-1 R | AGAAGATCATTCTCTGTGTTGGGA |  |

*Protospacer sequence is indicated in bold

**Table S3.**

Primers used in RT-qPCR

| **Gene** | **Sequence Forward (5’-3’)** | **Sequence Reverse (5’-3’)** |
| --- | --- | --- |
| *nsd3* | TCGGCAAACAAGAAACACAAG | AGATGGCTCTTGAGGTTGTG |
| *cel7a* | CCGAGCTTGGTAGTTACTCTG | GGTAGCCTTCTTGACTGAGT |
| *cel6a* | ACAAGAATGCATCGTCTCCG | TGTTCCACCCGTTGTAGTTG |
| *cel7b* | CCCTCAACACTAGCCACCAG | AGGTCTTGGAGGTGTCAACG |
| *cel3a* | CTGTACATCACCTACCCATC | TAGCTGAGATCTCGTCGTC |
| *cel1a* | TTTGCCTGGTCGCTCATG | AATCAGCTCGTCAAACAGCG |
| *xyn1* | GGCCAAATTATCGTCAACTGTC | TCTGTCTTTTGGGCTTGGAG |
| *xyn2* | TGTCAACGAGCCTTCCATC | TCTGCACAGTAACAGTTCCG |
| *bxl1* | CAAGTCTGGAATGAGGCTCTG | TGATGTCGGCAATCTGGTG |
| *chs2* | TGACCAGCAAAACTACGACTATG | CCGTCTTCCACCTCTTGATG |
| *chs4* | TCACAAAGGAGAATAGCAGACG | AGGATAAAATCAGGGCACCAG |
| *chs1* | TCAGGCAGTTTACGGACAAG | CAATGGTCGAGGTCATGGAG |
| *chs3* | CTGCTCAAGTATCTCTATCTCGC | AAGAACCAAGATGTAGCCCTG |
| *chs6* | GTTTGAAGGCATCGTGGTTAG | CGATCTTCCTCTCCCATGAAC |
| *chs5* | ACTTCCGCTACTTCACCAAC | CCCAGAGACTTGACGTACTTG |
| *chs7* | GTCCTACACCCAAGATCGTTC | TTCGTAGAGACCAGAGTAGACC |
| *chs8* | GACGAATTGACCCGCTCTC | CATTCTCGAAGTGACGGTACTG |
| *fks1* | CAGACAACCAGAAGCCTACC | GGAGAGGACCCATAAAGCATG |
| *tmk2* | CTTCCAGTCCTTCATCTACCAG | GCCAAAGTCGCAAATCTTCAG |
| *actin* | TGAGAGCGGTGGTATCCACG | GGTACCACCAGACATGACAATGTT |

**Table S4.**

Primers used in ChIP-qPCR

| **Gene** | **Sequence Forward (5’-3’)** | **Sequence Reverse (5’-3’)** |
| --- | --- | --- |
| *ace4* | GTCCGCTTCTGCTCCTTAG | GTCTACCAGAACCAGAACGG |
| *crt1* | CACTGATGCATGAAATTCGGC | CCACATGAAGAGACAAAGACAAAG |
| *stp1* | TCTGCTAGCGGTCTGGT | ACAGGCCTCGAGTATTCTCC |
| *cel3a* | GACATGACACTTGCCGTTTG | CCGGCAGTGATTCCTTGTATG |
| *cel1b* | CATCTGAATCAGCGAGGACC | TCCTGATCGAGTTCTTTGCC |
| *xyn2* | AGGTCATTGAATCCAGATCGG | AATTGGATATAATCGGACCCTGG |

**References**

1.	Silva-Rocha R, Castro LDS, Antoniêto ACC, Guazzaroni ME, Persinoti GF, Silva RN. 2014. Deciphering the cis-regulatory elements for XYR1 and CRE1 regulators in Trichoderma reesei. PLoS One 9.

2.	dos Santos Castro L, de Paula RG, Antoniêto ACC, Persinoti GF, Silva-Rocha R, Silva RN. 2016. Understanding the Role of the Master Regulator XYR1 in Trichoderma reesei by Global Transcriptional Analysis. Front Microbiol 7:1–16.

3.	Zhang J, Chen Y, Wu C, Liu P, Wang W, Wei D. 2019. The transcription factor ACE3 controls cellulase activities and lactose metabolism via two additional regulators in the fungus Trichoderma reesei. J Biol Chem 294:18435–18450.

4.	De Paula RG, Antonieto ACC, Carraro CB, Lopes DCB, Persinoti GF, Peres NTA, Martinez-Rossi NM, Silva-Rocha R, Silva RN. 2018. The Duality of MAPK Signaling Pathway in the Control of Metabolic Processes and Cellulases Production in Trichoderma reesei. Sci Rep manuscript submitted.
